# Large scale validation of an efficient CRISPR/Cas-based multi gene editing protocol in *Escherichia coli*

**DOI:** 10.1186/s12934-017-0681-1

**Published:** 2017-04-24

**Authors:** Francesca Zerbini, Ilaria Zanella, Davide Fraccascia, Enrico König, Carmela Irene, Luca F. Frattini, Michele Tomasi, Laura Fantappiè, Luisa Ganfini, Elena Caproni, Matteo Parri, Alberto Grandi, Guido Grandi

**Affiliations:** 1 Synthetic and Structural Vaccinology Unit, CIBIO, University of Trento, Via Sommarive, 9, Povo, 38123 Trento, Italy; 2 Toscana Life Sciences Scientific Park, Via Fiorentina, 1, 53100 Siena, Italy

**Keywords:** CRISPR-Cas9, High-throughput genome editing, Synthetic biology

## Abstract

**Background:**

The exploitation of the CRISPR/Cas9 machinery coupled to lambda (λ) recombinase-mediated homologous recombination (recombineering) is becoming the method of choice for genome editing in *E. coli*. First proposed by Jiang and co-workers, the strategy has been subsequently fine-tuned by several authors who demonstrated, by using few selected loci, that the *efficiency of mutagenesis* (number of mutant colonies over total number of colonies analyzed) can be extremely high (up to 100%). However, from published data it is difficult to appreciate the *robustness* of the technology, defined as the number of successfully mutated loci over the total number of targeted loci. This information is particularly relevant in high-throughput genome editing, where repetition of experiments to rescue missing mutants would be impractical. This work describes a “brute force” validation activity, which culminated in the definition of a robust, simple and rapid protocol for single or multiple gene deletions.

**Results:**

We first set up our own version of the CRISPR/Cas9 protocol and then we evaluated the mutagenesis efficiency by changing different parameters including sequence of guide RNAs, length and concentration of donor DNAs, and use of single stranded and double stranded donor DNAs. We then validated the optimized conditions targeting 78 “dispensable” genes. This work led to the definition of a protocol, featuring the use of double stranded synthetic donor DNAs, which guarantees mutagenesis efficiencies consistently higher than 10% and a robustness of 100%. The procedure can be applied also for simultaneous gene deletions.

**Conclusions:**

This work defines for the first time the robustness of a CRISPR/Cas9-based protocol based on a large sample size. Since the technical solutions here proposed can be applied to other similar procedures, the data could be of general interest for the scientific community working on bacterial genome editing and, in particular, for those involved in synthetic biology projects requiring high throughput procedures.

**Electronic supplementary material:**

The online version of this article (doi:10.1186/s12934-017-0681-1) contains supplementary material, which is available to authorized users.

## Background

*Escherichia coli* is one of the most extensively studied living organisms on earth and as such has become an instrumental model system for the understanding of a plethora of gene functions and regulations in both prokaryotes and eukaryotes. Moreover, *E. coli* also plays an invaluable role in modern biological engineering and industrial microbiology: it is a very versatile host for the production of heterologous proteins and their mass-production in industrial fermentation systems. Genetically modified *E. coli* cells are currently used in a wide range of processes, such as vaccine development, bioremediation, production of biofuels and production of immobilized enzymes. Additional biotechnological applications are expected to be developed thanks to the exploitation of modern techniques of metabolic engineering and synthetic biology.

To fully exploit the use of synthetic biology in *E. coli*, the availability of efficient genome editing systems, also applicable in high-throughput modalities is necessary. Currently, there are three main approaches for manipulation of chromosomal DNA in *E. coli*, all utilizing phage recombinase-mediated homologous recombination (recombineering), using either the Rac prophage system [1, 2] or the three bacteriophage λ Red proteins Exo, Beta, and Gam [3–5]. Classically, gene knockout mutants are created by inserting antibiotic resistance markers (or other selection markers) between double-stranded DNA (ds-DNA) PCR products derived from the upstream and downstream regions of the target gene. Mutant colonies are isolated in the appropriate selective medium after transformation with linear or circular constructs and, when necessary, the selection marker is subsequently eliminated by counter-selection, leaving a “scarless” chromosomal mutation. Court and co-workers elegantly demonstrated that chromosomal gene mutations can be achieved without the need of selection markers and using synthetic single stranded DNAs (ss-DNAs) or ds-DNAs, which anneal to their complementary chromosomal regions during replication and mediate recombination and gene modification [6, 7]. While effective, these two approaches are not ideal for high-throughput applications since they are laborious and time consuming (in the case of the first approach) and feature mutagenesis efficiencies often below 1% (in the case of the second approach) [8–10]. More recently, a third approach, proposed for the first time by Jiang and co-workers [9], makes use of the CRISPR/Cas9 technology [11–13]. Briefly, the strain to be modified is first genetically manipulated to express the Cas9 nuclease and the λ Red machinery, and subsequently the strain is co-transformed with (i) a plasmid (pCRISPR) encoding the guide RNA, which anneals with the chromosomal region to be modified and promotes a site-specific DNA cleavage by the Cas9, and (ii) a donor DNA (PCR-derived or chemically synthesized) partially homologous to the cleaved extremities, which promotes the repair of the double stranded break through λ Red-mediated recombination thereby introducing the desired mutation. The presence of the λ Red machinery plays an important role in the process since in its absence the mutagenesis efficiency was shown to drop quite substantially [10]. With this strategy, Jiang and co-workers reported mutation efficiencies as high as 65%. Subsequently, other authors fine-tuned the Jiang et al. protocol by adding innovative solutions and expanding its application for extensive gene deletions and replacements [14–17]. Thanks to the contribution of all these authors, CRISPR/Cas9 coupled to recombineering is becoming the most effective approach for genome editing in bacteria and in particular in *E. coli*.

However, one aspect of the CRISPR/Cas9 technology that still remains to be thoroughly addressed is the definition of its “*robustness*”. In fact, while it has been extensively demonstrated that the *efficiency of mutagenesis* (number of mutant colonies/total number of colonies analyzed) can be extremely high (close to 100%), the *robustness* (number of mutated loci/total number targeted loci) has not yet been defined experimentally in a rigorous manner. Knowing the robustness of the specific CRISPR/Cas-based protocol in use is particularly relevant in high-throughput applications, where the repetition of mutagenesis experiments and/or the analysis of large numbers of colonies would be impractical. There is a paucity of papers addressing the “robustness issue” and even in these works the number of targeted loci is limited. For instance, to evaluate the robustness of their protocol, Reisch and Prather [18] using an innovative two plasmid system, one expressing a tightly regulated Cas9 and the other the gRNA, demonstrated that point mutations could be introduced into two dispensable genes with efficiencies close to 100%. On the basis of these data the authors concluded that their system is robust enough to make point mutations (and also larger deletions) in any genomic location carrying an appropriate PAM site. To support their conclusion, the authors also claimed that using the same protocol they successfully inactivated five additional genes. Another recent study describes a CRISPR-based strategy that allowed the integration of entire metabolic pathways in seven distinct loci using ds-DNAs encoding homologous regions to the insertion site of different size with 75–100% efficiencies [19].

In the present paper, we present a “brute force” validation effort, which culminated in the definition of a highly robust, simple and rapid protocol for both single and multiple scarless chromosome manipulations. First, we tested several experimental conditions targeting four dispensable gene loci with the aim of investigating the parameters that mostly influence mutagenesis efficiency. On the basis of this analysis we defined a mutagenesis protocol, which we subsequently validated on a panel of 78 additional genes. The overall approach, which involved the construction of approximately one hundred pCRISPR plasmids, the execution of a few hundred transformation experiments and the analysis of several thousand colony PCR and sequencing, led to the definition of a high fidelity and rapid protocol, which guarantees the generation of single and multiple mutations of dispensable genes with a 100% confidence.

## Results

### Components of our CRISPR/Cas9 genome editing protocol

**Fig. 1:**
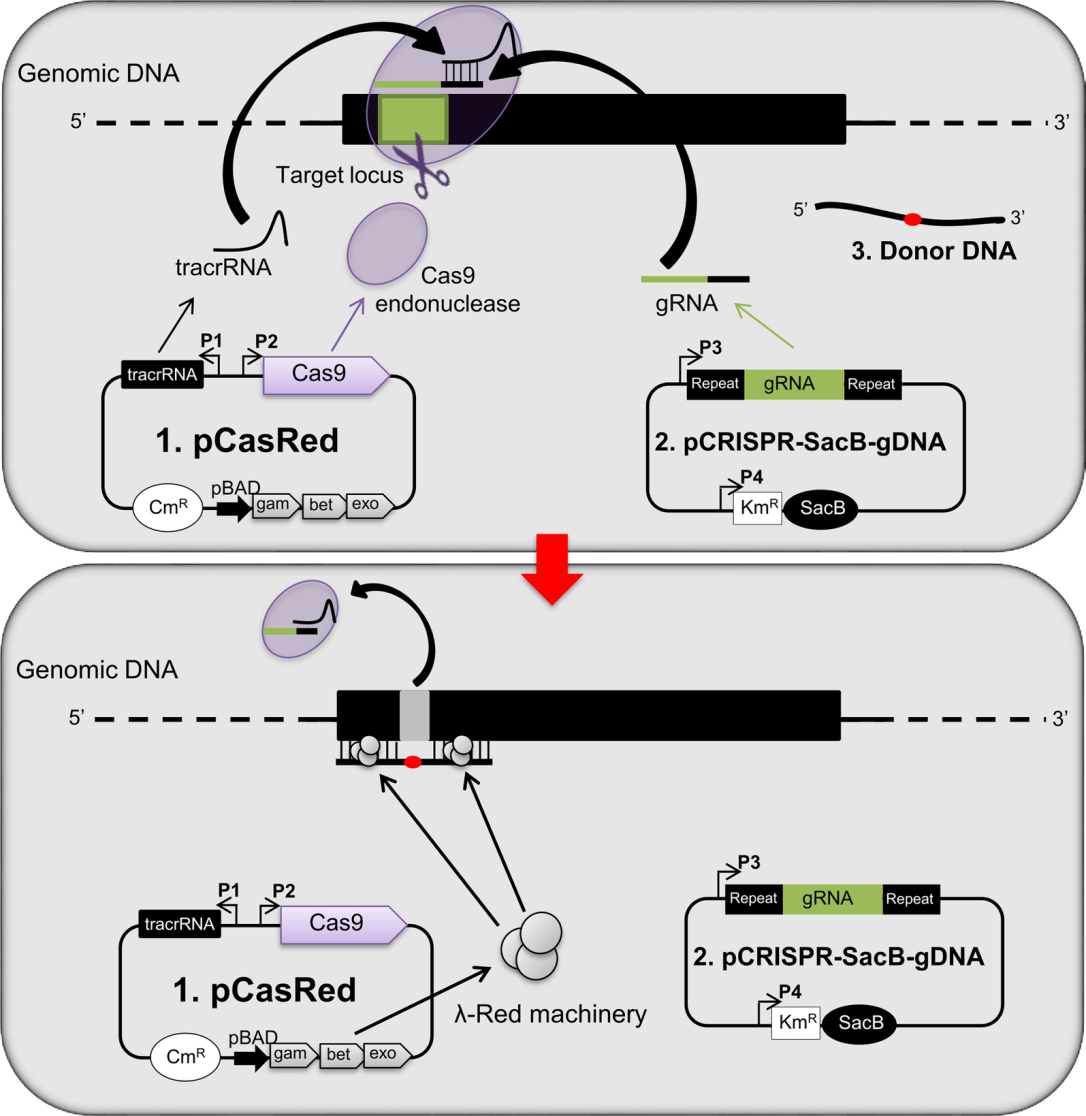
Overview of CRISPR/Cas9 genome editing strategy in *Escherichia coli*. The strain to be mutagenized [*E. coli* BL21(DE3)*∆ompA*] is first transformed with the pCasRed plasmid expressing the λ Red (Exo, Beta, Gam) machinery, the Cas9 endonuclease, and tracrRNA. Subsequently, the strain is co-transformed with pCRISPR-*SacB-gDNA*, and a synthetic, mutation-inducing oligonucleotide [donor DNA (dDNA)]. The pCRISPR-*SacB-gDNA* plasmid encodes the gRNA that specifies the site of cleavage and the endonuclease Cas9 recognizes the gRNA together with the tracrRNA, which anneals to gRNA forming a three-component complex. After the base pairing of gRNA to the target site, the Cas9 mediates the chromosomal DNA double strand break (*upper panel*). The double strand break is repaired by λ Red-mediated homologous recombination taking place between the extremities of the cleaved chromosomal DNA and the donor DNA (*lower panel*). For the sequence of constitutive promoters P1 and P2 see ADDGENE #4287 [9]; for sequence of constitutive promoter P3 see ADDGENE #42875 [9] and for P4 constitutive promoter sequence see ADDGENE #13036 [24]. For the arabinose-inducible promoter pBAD see pKOBEG plasmid [20]

Figure 1 schematically represents our CRISPR/Cas9-based *E. coli* genome editing protocol. Briefly, the procedure makes use of three main elements: the pCasRed plasmid, the pCRISPR-*SacB*-*gDNA* plasmid, and the synthetic, mutation-inducing oligonucleotide [donor DNA (dDNA)]. The pCasRed plasmid carries the chloramphenicol resistance gene (*Cm* ^*R*^) and encodes the Cas9 nuclease, the λ Red (Exo, Beta, Gam) cassette and the tracrRNA. The *cas9* gene and the tracrRNA coding sequence are under the control of constitutive promoters while the λ Red gene cassette is transcribed from the pBAD arabinose-inducible promoter [20]. The pCRISPR-*SacB*-*gDNA* plasmid derives from a pCRISPR plasmid [9], where the kanamycin resistance gene (*Km* ^*R*^) is fused to the *sacB* gene encoding the *Bacillus subtilis* levansucrase. *SacB *is toxic in *E. coli* if grown in media containing 5% sucrose [21–24] and, as previously demonstrated by Hale and co-workers, plasmid-borne sucrose toxicity can be exploited to cure high copy number plasmids [25]. We used this strategy to remove the pCRISPR-*SacB*-*gDNA* plasmid after the gene mutation has been introduced. In addition to the *sacB* gene, pCRISPR-*SacB*-*gDNA* plasmid carries the synthetic DNA fragment (gDNA), transcribed from a constitutive promoter [9], encoding the RNA guide necessary to drive the Cas9-dependent double stranded break to the desired site within the bacterial genome.

In the following sections we provide details of the experimental data that led us to define a protocol, which, under the conditions described here, guarantees the inactivation of any dispensable gene and, after construction of the pCRISPR plasmids and dDNAs (see “Methods”), allows the sequential mutation of genes at a pace of one mutation every other day. Finally, we also describe a highly efficient protocol for the simultaneous introduction of multiple mutations.

### Definition of the robustness of gDNA selection method

gDNAs within a target gene are usually selected among those 30 nucleotide sequences followed by an NGG (PAM) trinucleotide, which do not share homologies with other regions in the chromosome (to avoid off-target cleavage). Routine bioinformatics tools (BLAST) are generally used to identify such sequences. However, what has not been fully investigated is the robustness of bioinformatics in gDNA selection. In other words, do all predicted gDNAs promote an efficient CRISPR/Cas9-mediated cleavage at the selected target site? To test this, we selected several gDNAs and we analyzed their capacity to promote Cas9 cleavage. Theoretically, if a gDNA efficiently drives Cas9 cleavage no colonies should be isolated since non-homologous end-joining repair (NHEJ) works poorly in *E. coli* [26, 27]. Practically, colonies are still recoverable and represent “escapers” in which the gDNA from pCRISPR-*SacB*-*gDNA* is lost, or mutations in the PAM region and/or in the seed region (8–12 nucleotides upstream from the PAM region) [9] have occurred. Alternatively, as suggested by Cui and Bikard [28], if Cas9-mediated cleavage is not efficient, homologous recombination between the cleaved chromosome and the un-cleaved sister chromosome could take place. Finally, gDNA promoter mutations and mutations to the *cas9* gene itself could occur, leading to a basal level of colonies lacking of dsDNA breaks. However, whatever mechanism is involved, the number of “escaper” colonies should be orders of magnitude lower compared to the colonies obtained by transforming the strain with the “empty” pCRISPR-*SacB* plasmid. Therefore, the drop in transformation efficiency between pCRISPR-*SacB* and pCRISPR-*SacB*-*gDNA* indicates the quality of the gDNA.

**Fig. 2:**
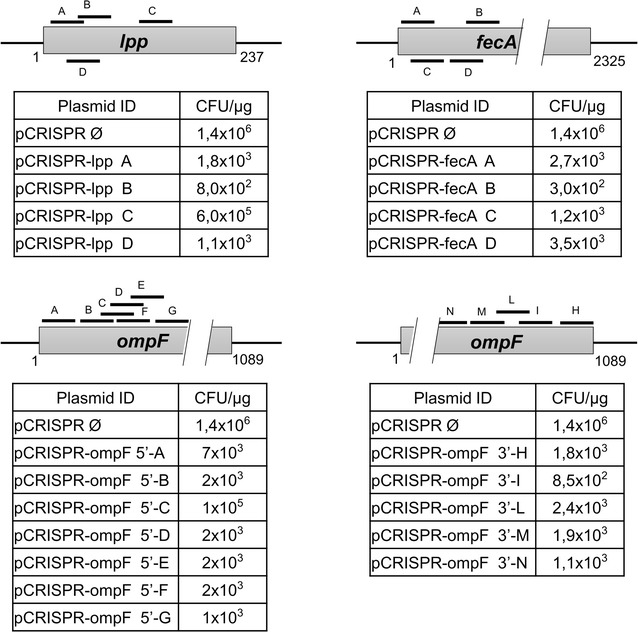
Selection of gDNAs for mutation of *ompF*, *lpp* and *fecA* genes. The *grey bars* are a schematic drawing of the genes *lpp*, *fecA* and *ompF*, and the *black lines* labelled with letters indicate the positions where the gRNAs transcribed from their corresponding gDNAs hybridize within each gene. gDNAs were cloned into pCRISPR, generating the plasmids reported in the Additional file 1: Table S1. The *tables* report the transformation efficiencies (CFU/μg) of each pCRISPR-*gDNA* in BL21(DE3)*∆ompA*(pCasRed)

To establish the effectiveness of gDNAs in our protocol, we created sets of pCRISPR-*gDNA* plasmids (Additional file 1: Table S1) by cloning synthetic gDNAs (Additional file 1: Table S2) between the two tracrRNA-complementary repeat regions (Fig. 1) and we compared the transformation efficiencies of the recombinant plasmids with the efficiency of the “empty” pCRISPR vector (Fig. 2). In particular, we selected three target genes, *ompF*, *lpp* and *fecA*, and we designed four sets of different gDNAs, two of them targeting different sites at the 5′ and 3′ regions of *ompF* gene, respectively, and the other two sets targeting *lpp* and *fecA* at different positions (Fig. 2). Overall, 20 pCRISPR-*gDNA* plasmids were generated and used to transform *E. coli* BL21(DE3)Δ*ompA*(pCasRed). The BL21(DE3)Δ*ompA* was used since we observed that the strain maintained good transformation efficiencies even in the presence of pCasRed plasmid. Colonies were selected on LB plates supplemented with Cm and Km. While the “empty” pCRISPR vector routinely gave a transformation efficiency of 0.5–2 × 10^6^ CFUs/μg of plasmid DNA, most of pCRISPR-*gDNA* plasmids had transformation efficiencies in the range of 0.5–2 × 10^3^ CFUs/μg of plasmid DNA (Fig. 2). However, two out of the 20 pCRISPR-*gDNA* plasmids (pCRISPR-*ompF_5'C* and pCRISPR-*lpp_C*) gave a transformation efficiency close to the one observed with the empty vector.

We randomly analyzed ten clones from the transformations with pCRISPR-*ompF_5′G* and pCRISPR-*ompF_5′C* (transformation efficiency of 1 × 10^3^ and 1 × 10^5^ CFUs/μg, respectively) using PCR and sequence analyses of both the chromosomal regions targeted by the corresponding gDNAs and the gDNA regions of pCRISPR-*ompF_5′G* and pCRISPR-*ompF_5′C*. While the chromosome target sites had wild type sequences in both sets of transformant colonies, the colonies transformed with pCRISPR-*ompF_5′G* carried rearranged plasmids missing the gDNA ompF_5′G region, probably due to homologous recombination occurring between the two repeats flanking the gDNA. By contrast, all the colonies analyzed from the transformation with pCRISPR-*ompF_5′C* carried a wild type gDNA sequence (Additional file 2: Figure S1) [9].

From these experiments we conclude that in silico selection allows the identification of gDNAs, which in most cases drive the Cas9-mediated cleavage with high efficiency. However, approximately 10% of selected gDNAs did not cause bacterial killing. “Good and bad” gDNAs can easily be discriminated by colony counting after transformation with the pCRISPR plasmids carrying the selected gDNAs. Therefore, before modifying any target gene we routinely run the colony testing and if transformation efficiencies higher than 10^3^ CFUs/μg of plasmid DNA is obtained we change the gDNA sequence of the plasmid.

### Definition of the optimal conditions to be used in gene knockout

Next, we investigated the efficiency of gene inactivation using synthetic oligonucleotides (donor DNA, dDNA) under different experimental conditions.

We analyzed three parameters: length and concentration of the mutagenic dDNA and extent of gene deletion. These parameters were first tested targeting the 5′ end of the *ompF* gene using the pCRISPR-*ompF_5′G* plasmid (Fig. 2) and single stranded donor DNAs annealing to the lagging strand (Lg-ss-dDNAs) (Additional file 1: Table S3). In fact, similar to the homologous recombination using synthetic oligonucleotides [5], Lg-ss-dDNAs have been reported to be more efficient in promoting double strand break repair than those targeting the leading strand (Ld-ss-dDNAs) [10, 18]. Donor DNAs were designed with homology arms of equal length upstream and downstream from the deletion. Subsequently, the best conditions were validated targeting three additional gene loci, the 3′ end of the *ompF* gene, and the *fecA* and *lpp* genes, using either single stranded donor DNA (Ld-ss-dDNA) or double stranded donor DNA (ds-dDNA). All parameters were tested in at least three independent experiments and mutation efficiencies were determined by analyzing a total of at least 20 colonies.

Table 1 summarizes the data obtained from all experiments. As far as the use of ss-dDNA is concerned, for short gene deletions (around 30 bp), 10 μg of both 70 and 120 bp oligonucleotides were very effective, resulting in efficiencies of mutagenesis >50% for all four genes. Deletions longer than 30 bp and up to approximately 500 bp, could be obtained with relatively good efficiencies (>10%) as long as 10 μg of 120 bp ss-dDNAs were used. However, efficiencies dropped quite substantially (<5%) for deletions longer than 500 bp. No major appreciable difference in deletion efficiencies at all four gene loci was observed when the Ld-ss-dDNA was used in place of the Lg-ss-dDNA. As far as ds-dDNA is concerned, the use of 120 bp ds oligonucleotides was not only as efficient as ss-dDNAs for short deletions but also allowed the generation of the long full gene deletions that failed using ss-dDNAs.

**Table 1:** Influence of type of donor DNA (dDNA) (Lg-ss-dDNAs, Ld-ss-dDNAs, ds-dDNA) length of dDNA, concentration of dDNA and size of deletion on mutagenesis efficiency at four chromosomal loci

Target gene-pCRISPR-gDNA	Type of dDNA	dDNA ID	dDNA length (nt)	Mutation (nt)	dDNA quantity (µg)	Efficiency (%)-positive/total
ompF 5′-pCRISPR-ompF_5′G	ss-Lg	ompF_5′G-70-∆30	70	∆30	1	86% (19/22)
			70	∆30	10	95% (40/42)
		ompF_5′G-70-∆100	70	∆100	10	5% (2/36)
		ompF_5′G-70-∆500	70	∆500	10	0% (0/30)
		ompF_5′G-120-∆30	120	∆30	1	64 ± 34% (40/62)
			120	∆30	10	93% (76/82)
		ompF_5′G-120-∆100	120	∆100	10	64 ± 31% (20/31)
		ompF_5′G-120-∆500	120	∆500	10	47 ± 19% (14/30)
		ompF_5′G-120-∆1089	120	∆1180	10	0% (0/20)
	ss-Ld	ompF_5′G-70-∆30 R	70	∆30 nt	10	20% (4/20)
		ompF_5′G-120-∆30 R	120	∆30	10	100% (10/10)
		ompF_5′G-120-∆1089 R	120	∆1180	10	0% (0/> 100)
	ds	ompF_5′G-120-∆30 ds	120	∆30	10	77% (20/26)
		ompF_5′G-120-∆500 ds	120	∆500	10	50 ± 14% (15/30)
		ompF_5′G-120-∆1089 ds	120	∆1180	10	26 ± 17% (6/23)
ompF 3′-pCRISPR-ompF_3′I	ss-Lg	ompF_3′I-70-∆30	70	∆30	10	79% (19/24)
		ompF_3′I-120-∆30	120	∆30	10	83% (25/30)
fecA-pCRISPR-fecA_B	ss-Lg	fecA_B-70-∆30	70	∆30	10	43 ± 4% (13/30)
		fecA_B-120-∆30	120	∆30	10	77% (23/30)
		fecA_B-120-∆100	120	∆100	10	20% (6/30)
		fecA_B-120-∆500	120	∆500	10	13% (4/30)
		fecA_B-120-∆2325	120	∆2325	10	0% (0/30)
	ds	fecA_B-120-∆2325 ds	120	∆2325	10	14% (5/35)
lpp-pCRISPR-lpp_B	ss-Lg	lpp_B-70-∆30 R	70	∆30	10	20% (4/20)
		lpp_B-120-∆30 R	120	∆30	10	55 ± 7% (11/20)
		lpp_B-120-∆237 R	120	∆237	10	0% (0/16)
	ss-Ld	lpp_B-70-∆30	70	∆30	10	90% (27/30)
		lpp_B-120-∆30	120	∆30	10	73% (19/26)
		lpp_B-120-∆237	120	∆237	10	0% (0/30)
	ds	lpp_B-120-∆30 ds	120	∆30	10	82% (19/23)
		lpp_B-120-∆237 ds	120	∆237	10	72% (18/25)

From the experimental data obtained on four gene loci, we conclude that, in order to create relatively short deletions, ss-dDNAs of 70–120 nucleotides targeting either the lagging or the leading strands perform well (100% robustness and >50% efficiency). However, 120 bp-ds-dDNAs showed high robustness and efficiency when extended deletions (of up to 2.200 bp) were required.

### Validation of 30 bp gene deletions for mutant library production

**Fig. 3:**
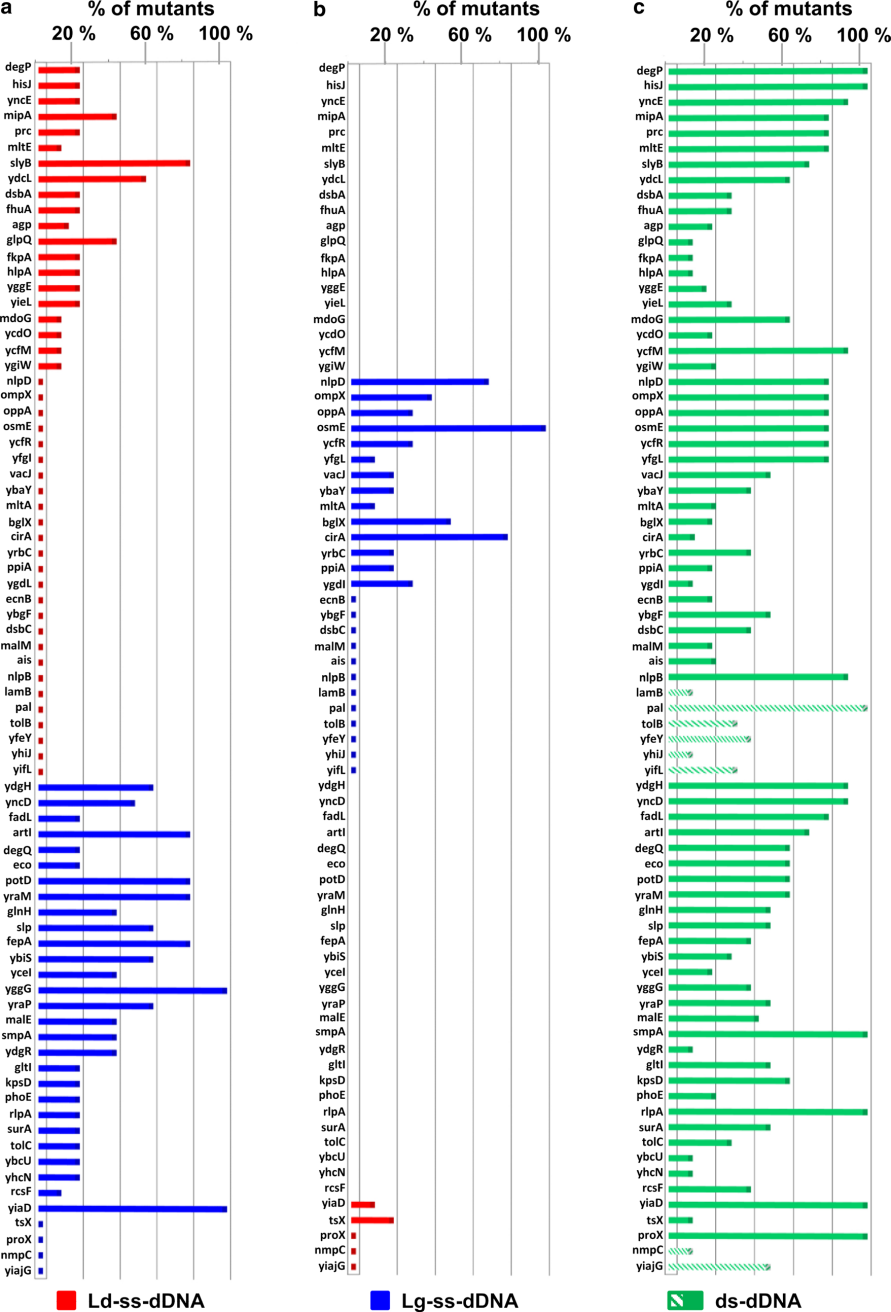
Validation of 30 bp deletions on 78 genes. *E. coli* BL21(DE3)Δ*ompA*(pCasRed) or *E. coli* BL21(DE3)(pCasRed) were transformed with different mixtures of pCRISPR-*SacB-gDNAs* and 70-base dDNAs (either ssDNA or dsDNA) to mediate 30 bp deletion at one of the 78 selected gene loci (*y axis*). Ten colonies from each transformation were analyzed by PCR to identify those carrying the deletion. *X axis* indicates the percentage of mutants identified out of the total number of colonies analyzed. *Bar height* indicates the mutation frequency, while the presence of *flat colored squares* above gene names in each graph indicates that no mutants were identified out of ten colonies analyzed. *Absence of bars* or *flat colored squares* above gene names indicate that the gene mutation was not attempted for those specific genes in the experiment indicated in each bar graph. *Red bars/squares* indicate mutation experiments using Ld-ss-dDNAs; *Blue bars/squares* indicate mutation experiments using Lg-ss-dDNAs; *Green bars/squares* indicate mutation experiments using ds-dDNAs; *bars with green downward diagonals* indicate mutation experiments with ds-dDNAs in BL21(DE3)(pCasRed). **a** *Bar graph* representing mutation success rate using 46 Ld-ss-dDNAs (*red bars/squares*) and 32 Lg-ss-dDNAs (*blue bars/squares*). **b** Gene mutations that failed using the Ld-ss-DNAs (26 genes out of 46) and the Lg-ss-DNAs (4 genes out of 32) were re-attempted using ss-dDNAs targeting the opposite strands. The *chart* represents the mutation success rate of this second round of experiments. **c** The *bar graph* represents the mutation success rate in BL21(DE3)*ΔompA*(pCasRed) (*green bars*) and in BL21(DE3)(pCasRed) (*green downward diagonal bars*) using ds-dDNAs

To further validate the conclusions of the experiments described above, we attempted the mutation of 78 additional genes that are classified as “dispensable” according to the Keio library [29] (see Additional file 1: Table S4 for the list of genes). First, we constructed the 78 pCRISPR-*SacB*-*gDNAs* plasmids (Additional file 1: Table S1) encoding the gRNAs targeting the genes next to their 5′ ends to avoid the expression of truncated but nonetheless functional proteins (Additional file 1: Table S2). Subsequently, we verified the effectiveness of the cloned gDNAs in guiding Cas9 cleavage by transformation and colony counting (see above). In line with the statistics previously obtained, approximately 10% of selected gDNAs (7 out of 78, highlighted with an asterisk in Additional file 1: Table S2) did not promote Cas9 cleavage, and therefore other functional gDNAs were selected (Additional file 1: Table S2). After selecting the 78 pCRISPR-*SacB*-*gDNAs* plasmids with transformation efficiencies three logs lower than pCRISPR-*SacB*, we next designed 78 70 bp-ss-dDNAs to delete 30 bp and introduce a premature stop codon in each gene, downstream the deleted region (Additional file 1: Table S3). Of these 78 dDNAs, 46 targeted the leading strand and 32 the lagging strand. Each pCRISPR-*SacB*-*gDNA* plasmid, with its corresponding dDNA, was used to transform *E. coli* BL21(DE3)Δ*ompA*(pCasRed) strain, giving transformation efficiencies usually between 2 and 5 × 10^2^ CFUs/μg of plasmid DNA. From each transformation ten colonies were randomly selected and analyzed by PCR to identify those carrying the 30 bp deletion and to establish the “success rate” of mutagenesis. We arbitrarily applied the “ten colony rule” (i.e., mutagenesis efficiencies higher that 10%) because we considered such number still compatible with manual high-throughput procedures. Figure 3a summarizes the results of our screening. Forty-eight of the selected 78 genes were successfully mutated, with efficiencies ≥10% (at least one mutant/10 colonies). More specifically, 20 of the 46 Ld-ss-dDNAs created the desired mutations, while Lg-ss-dDNAs mutated 28 out of 32 genes.

The inability to obtain 30 mutations, at least with a sufficiently high frequency to fulfill the “ten colony rule”, was partially unexpected, since the missing mutations had been previously obtained in *E. coli* K-12 BW25113 [29]. Therefore, either such mutations are not compatible with the genetic background of our recipient strain [our strain is an *E. coli* BL21(DE3) derivative carrying the deletion of the *ompA* gene and pCasRed plasmid] or the experimental conditions previously defined were not sufficiently robust.

To discriminate between the two possibilities, we first asked the question whether the use of dDNAs targeting the opposite strands could rescue the missing mutants. Figure 3b reports the results of this experiment. The use of Lg-ss-dDNA rescued 14 of the 26 mutants that failed to be isolated with the Ld-ss-dDNA. In contrast, when the mutation of the five genes that were not mutated with the Lg-ss-dDNA was attempted only one mutant was rescued using Ld-ss-dDNA. Overall, the data indicate that 42 out of a total of 58 mutagenesis attempts with the Lg-ss-dDNA were successful, corresponding to a success rate of 72%. By contrast, the use of the Ld-ss-dDNA was successful in 22 of the 51 mutagenesis experiments (success rate 43%).

These data demonstrate that, differently from our preliminary validation experiment but in line with previous studies [10, 18], Lg-ss-dDNA performed better than Ld-ss-dDNA both in terms of success rate (72% rate versus 43%) and efficiency of mutagenesis (average bar heights with Ld-ss-dDNA: 10%; average bar heights with Lg-ss-dDNA: 31%). However, a non-negligible fraction of genes [15 out of 78 (19%)] failed to be mutagenized, regardless the type of the dDNA used.

Considering our experimental data that showed how ds-dDNAs successfully guided long deletions not obtained with ss-dDNAs, we thus re-attempted the deletion of all 78 genes using the same pCRISPR-*SacB*-*gDNAs*, but making the 70 base dDNAs double stranded. As shown in Fig. 3c, all genes that were successfully mutated using either the Lg-ss-dDNAs or the Ld-ss-dDNAs (63 genes in total) were also inactivated with the ds-dDNA. In addition, seven genes that were not deleted with the ss-dDNAs were mutagenized, bringing the success rate to 90% (70/78). Importantly, when ds-dDNAs were used the mutagenesis efficiency was also improved compared to the efficiencies obtained with ss-dDNAs (average bar heights with ds-dDNA: 48.9%).

As pointed out above, the inability to obtain some mutations classified as “dispensable” according to the Keio library [29] could be due to the fact that such mutations are not compatible with the genetic background of our recipient strain [our strain is an *E. coli* BL21(DE3) derivative carrying the deletion of the *ompA* gene and pCasRed plasmid]. To investigate the possible role of the Δ*ompA* deletion in preventing the inactivation of the eight genes, we tested whether they could be achieved in BL21(DE3) wild type strain. To this aim, pCasRed plasmid was introduced into BL21(DE3) and BL21(DE3)(pCasRed) was subsequently subjected to the gene deletion experiments by co-transforming the strain with the eight pCRISPR/ds-dDNAs couples. As a control, the deletion of the *degP* and *yncD* genes [two genes that were successfully inactivated in BL21(DE3)Δ*ompA* was also attempted]. As shown in Fig. 3 and Additional file 1: Table S6, all eight genes were successfully inactivated, with good efficiencies, thus confirming the incompatibility of these mutations with the Δ*ompA* gene inactivation. It is noteworthy that all the eight genes encode membrane-associated proteins whose inactivation in the absence of one of the major *E. coli* outer membrane proteins might impair the membrane function and/or structural integrity. An important outcome of the mutagenesis experiments carried out in BL21(DE3) strain is that when ds-dDNA is used, our CRISPR/Cas9 mutagenesis protocol can efficiently inactivate any “dispensable” gene and therefore the protocol has a robustness of 100%.

### Creation of multi-gene mutants by stepwise approach

**Fig. 4:**
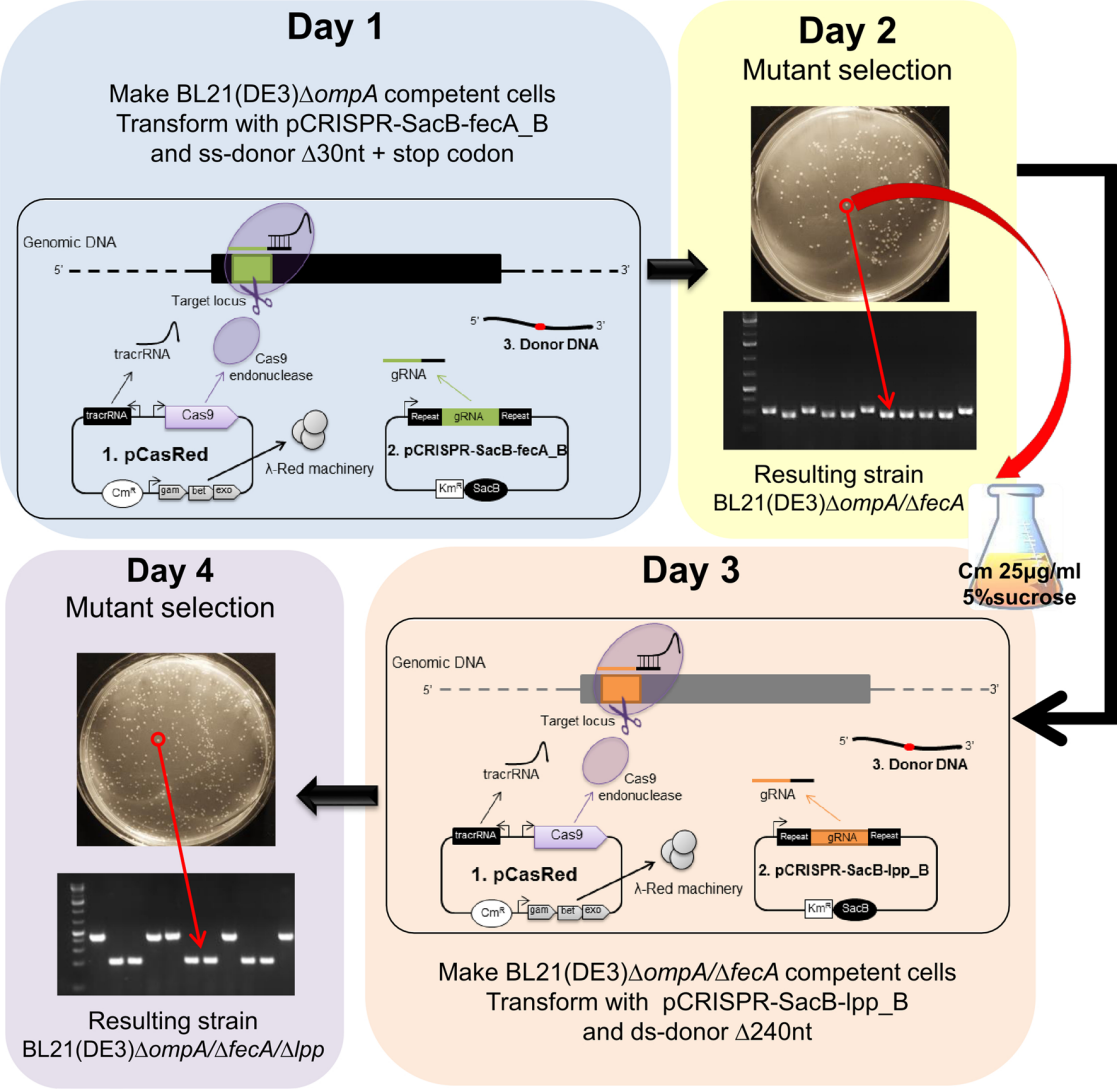
Representation of the stepwise approach used to isolate strains carrying multiple mutations. Day 1: *E. coli* BL21(DE3)*∆ompA*(pCasRed) was co-transformed with 1 μg/ml of pCRISPR-*SacB-fecA_B* and 10 μg/ml of donor fecA-120-∆30nt and transformant colonies were selected on LB agar plates supplemented with Cm (25 μg/ml) and Km (50 μg/ml). Day 2: Ten colonies were randomly selected and screened by PCR using primers designed to generate DNA fragments from mutated colonies of 200 bp. PCR products were analyzed on 2% agarose gels. One mutant clone was subsequently inoculated into 5 ml of LB supplemented with 5% sucrose and 25 μg/ml Cm. Day 3: The overnight culture was used to prepare competent cells, which were subsequently co-transformed with 1 μg/ml of pCRISPR-*SacB-lpp_B* and 10 μg of double strand donor DNA lpp-120-Δ237. Day 4: Ten colonies were randomly selected and screened by PCR using primers designed to generate DNA fragments from mutated colonies of 400 bp. PCR products were analyzed on 2% agarose gels

In order to generate multi-gene mutants in a stepwise modality, the pCRISPR-*gDNA* plasmid used to knockout a given gene has to be removed to allow the next round of transformation with a pCRISPR-*gDNA* plasmid targeting another gene locus. The pCRISPR plasmid is a high copy number plasmid (>400 copies/cell in exponential growth phase) and therefore it cannot be easily removed by simply growing bacteria in liquid media deprived of kanamycin, the pCRISPR antibiotic resistance marker. Elegant solutions have been proposed to get rid of high copy number plasmids [10, 18, 30]. We adopted the strategy proposed by Hale and co-workers [25] by introducing the suicide *sacB* gene downstream of the Km cassette into the pCRISPR plasmid, thereby generating the pCRISPR-*SacB* plasmid. *E. coli* strains carrying any pCRISPR-*SacB* plasmid derivative can survive in the presence of sucrose only if they rapidly lose the plasmid [21–24]. The efficiency of pCRISPR-*SacB* plasmid elimination in sucrose containing media can accelerate the entire process of multiple stepwise gene inactivation. Once a mutant colony is selected by colony PCR, it can be inoculated in sucrose-containing LB and the overnight culture can be directly used to make competent cells for the subsequent mutagenesis step. We validated the procedure by creating a BL21(DE3)Δ*ompA*(pCasRed) strain carrying the inactivation of both *fecA* and *lpp* genes. First of all, the gDNA *fecA_B* and gDNA *lpp*_B were inserted into pCRISPR-*SacB* plasmid generating plasmids pCRISPR-*SacB*-*fecA*_B and pCRISPR-*SacB*-*lpp*_B plasmids (Additional file 1: Table S1), respectively. Next, BL21(DE3)Δ*ompA*(pCasRed) strain was co-transformed with pCRISPR-*SacB*-*fecA*_B plasmid and the 120 base oligo fecA_B-120-∆30 to create the 30 bp *fecA* gene deletion. Seven out of the ten colonies selected on Km/Cm LB plate carried the expected mutation (Fig. 4). One mutant clone was grown overnight in LB supplemented with Cm and 5% sucrose and competent cells, directly prepared from the overnight culture, were co-transformed with pCRISPR-*SacB*-*lpp*_B plasmid and the double stranded 120 base lpp_B-120-∆240 oligo designed to eliminate the entire *lpp* gene. BL21(DE3)*ompA/*Δ*fecA/*Δ*lpp* colonies were obtained with an efficiency of 60% (Fig. 4). By the time the two pCRISPR-*SacB*-*gDNA* plasmids and the two dDNAs were available, four working days were sufficient to generate the BL21(DE3)Δ*ompA* derivative strain carrying the Δ*fecA* and Δ*lpp* double gene deletions.

### Simultaneous creation of multi-gene deletions

**Fig. 5:**
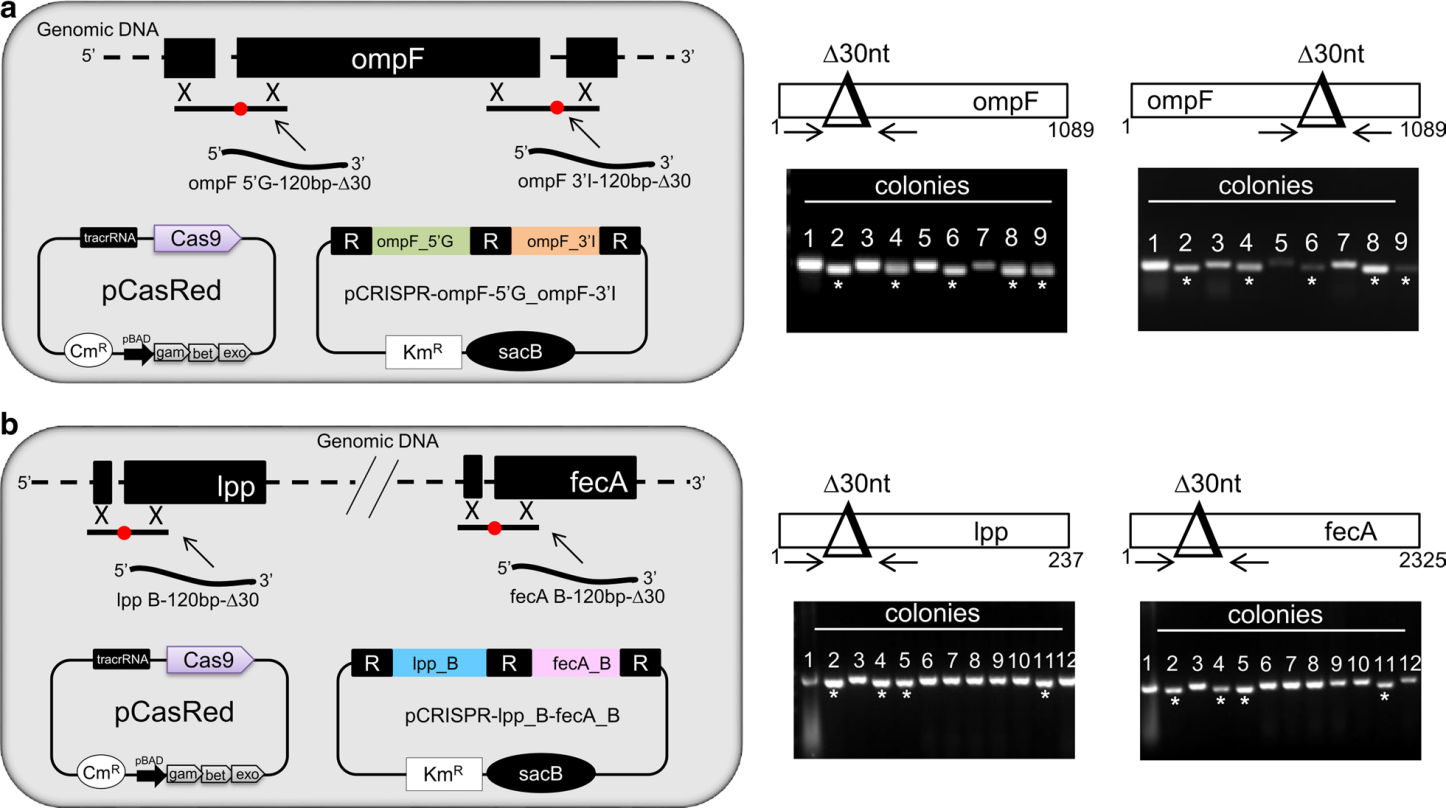
CRISPR/Cas-based protocol for simultaneous two-gene deletions *E. coli* BL21(DE3)*∆ompA*(pCasRed) was co-transformed with either 100 ng pCRISPR-*ompF_5′G*-*ompF_3′I* plasmid and the two dDNAs ompF_5′G-120-∆30 and ompF_3′I-120-∆30 (10 μg each) (**a**) or with 100 ng pCRISPR-*lpp_B*-*fecA_B* and lpp_B-120-∆30 and fecA_B-120-∆30 dDNAs (10 μg each). **b** Transformant colonies were selected on LB agar plates supplemented with 25 μg/ml Cm and 50 μg/ml Km. Colony PCR was carried out using two different couple of primers to screen each genomic locus (indicated at the *bottom* of each gel) on a randomly selected number of colonies and the PCR products separated on 2% agarose gels. *Asterisks* indicate those colonies in which deletion occurred at both gene loci. The primer sequences used for PCR experiments are reported in Additional file 1: Table S5

Finally, we tested the possibility of exploiting the CRISPR/Cas technology to simultaneously create mutations at two separated genome loci. To test this strategy, we created pCRISPR plasmids carrying two gDNAs designed to guide the Cas-mediated genome cleavage at two distant positions within the *E. coli* BL21(DE3*)*Δ*ompA* genome. The two gDNAs were intercalated by the short repeated regions in the configuration “Repeat-gDNA_1_-Repeat-gDNA_2_-Repeat” (Additional file 1: Table S1; Fig. 5). We mimicked the same organization of the CRISPR arrays found in bacteria. The pCRISPR(gDNA)_2_ plasmid thus obtained was co-transformed with two mutagenic oligonucleotides designed to repair and mutagenize the genome at the two cleaved sites.

First, we tested this approach by creating pCRISPR-*ompF_5′G*-o*mpF_3′I* plasmid to delete 30 bp at both the 5′ and 3′ ends of the *ompF* gene (Fig. 5a). To verify that the plasmid carrying two guide RNAs could drive the simultaneous Cas-mediated cleavage at two chromosomal sites, *E. coli* BL21(DE3*)*Δ*ompA*(pCasRed) strain was transformed with pCRISPR-*ompF_5′G*-*ompF_3′I* plasmid in the presence of either ompF_5′G-120-∆30 or ompF_3′I-120-∆30 donor DNAs. If pCRISPR-*ompF_5′G*-*ompF_3′I* mediates the Cas9 cleavage of the *ompF* gene at both sites, co-transformation with only one dDNA should not generate mutants. Indeed, co-transformation of pCRISPR-*ompF_5′G*-*ompF_3′I* with ompF_3′I-120-∆30 donor DNA gave no mutants (from 20 colonies tested) and the co-transformation of pCRISPR-*ompF_5′G*-*ompF_3′I* with ompF_5′G-120-∆30 dDNA gave only two mutants out of 20 screened colonies (Table 2). Next, we co-transformed BL21(DE3*)*Δ*ompA*(pCasRed) with pCRISPR-*ompF_5′G*-*ompF_3′I* in the presence of both ompF_5′G-120-∆30 and ompF_3′I-120-∆30 dDNAs and we analyzed 20 colonies by PCR. Twelve out of 20 colonies carried the deletion at both sites while only one of the 20 colonies analyzed carried a single mutation at the 5′ end of the *ompF* gene (Fig. 5; Table 2).

**Table 2:** Efficiency of simultaneous two-loci mutagenesis (ompF 5′/ompF 3′ regions and fecA/lpp genes) using pCRISPR plasmids carrying REPEAT-gDNA_1_-REPEAT-gDNA_2_-REPEAT cassette

pCRISPR-gDNA	dDNA ID	Mutation		
pCRISPR-ompF-5′G_ompF-3′I	ompF_5′G-120-∆30	*∆30 ompF_5′*	*∆30 ompF_3′*	*∆30 ompF_5′*/*∆30 ompF_3′*
		10% (2/20)	Not tested	Not tested
pCRISPR-ompF-5′G_ompF-3′I	ompF_3′I-120-∆30	*∆30 ompF_5′*	*∆30 ompF_3′*	*∆30 ompF_5′*/*∆30 ompF_3′*
		Not tested	0% (0/20)	Not tested
pCRISPR-ompF-5′G_ompF-3′I	ompF_5′G-120-∆30 + ompF_3′I-120-∆30	*∆30 ompF_5′*	*∆30 ompF_3′*	*∆30 ompF_5′*/*∆30 ompF_3′*
		5% (1/20)	0% (0/20)	57% (12/20)
pCRISPR-lpp_B-fecA_B	lpp_B-120-∆30 + fecA_B-120-∆30	*∆30 lpp*	*∆30 fecA*	*∆30 lpp*/*∆30 fecA*
		3% (1/29)	0% (0/29)	31% (9/29)

To confirm the applicability of the simultaneous double gene knockout, we also tested the concomitant inactivation of *lpp* and *fecA* genes. For this purpose, pCRISPR-*lpp_B*-*fecA_B* was constructed (Fig. 5b) and used to co-transform *E. coli* BL21(DE3*)*Δ*ompA*(pCasRed) with lpp_B-120-∆30 and fecA_B-120-∆30 donor DNAs (Table 2). Here, nine out of 29 colonies were mutated in both genes, while single gene mutations were obtained at frequencies of 0% for *fecA *and 3% for *lpp *(Fig. 5b; Table 2).

## Discussion

The cutting edge CRISPR/Cas-technology was elegantly applied to bacteria for the very first time by Jiang and co-workers in 2013 [9]. Subsequently, a number of other groups, using modified versions of the protocol proposed by Jiang et al. demonstrated the broad applicability and flexibility of the technology in several bacterial species. Thanks to these discoveries, the CRISPR/Cas methodology became the most effective strategy to create gene knock-outs and knock-ins in prokaryotes (for a recent review see Choi and Lee [14]). Two are the major breakthroughs of CRISPR/Cas-technology. First, the efficiency of mutagenesis can be as high as 100%. As reported by several authors [9, 10], such efficiency cannot be reached by any other genome editing strategy, including the procedure based on the λ Red/synthetic oligonucleotide-mediated homologous recombination described by Court and co-workers. For instance, when we tested the efficiency of *ompF* 30 bp deletion transforming BL21(DE3)*ΔompA*(pCasRed) with the synthetic dDNA in the absence of the corresponding pCRISPR-*gDNA*, we did not manage to isolate a single mutant colony out of 40 colonies analyzed. Second, even large deletions can be achieved at high frequencies using synthetic oligonucleotides as donor DNAs, thus avoiding the need to generate linear or circular DNA constructs to drive homologous recombination.

However, one missing information from published data was the robustness of the CRISPR/Cas protocols. In other words, how confident could one be that any desired mutation can be consistently achieved at high frequency? This information becomes particularly relevant when CRISPR/Cas genome editing is applied in high-throughput formats where the screening of several colonies and the repetition of mutagenesis experiments to rescue missing mutants could be impractical.

To address this issue, we first set up our own version of CRISPR/Cas9-based genome editing and we defined the best conditions by analyzing the mutagenesis efficiency at four gene loci using different parameters, including (i) sequence of the guide RNA (ii) length and concentration of the dDNA, and (iii) use of ss-dDNA and ds-dDNAs. Subsequently, we challenged the robustness of the protocol by attempting 30 bp deletion in 78 additional genes selected on the basis of their being classified as “dispensable” according to the Keio collection [29]. In this way, and for the first time, we could assign a value to the success rate of a CRISPR/Cas9-based genome editing procedure based on a statistically meaningful number of data (81 target loci in total).

The relevant outcome from our work is that we could establish with a high degree of confidence that our CRISPR/Cas9 protocol features an efficiency of mutagenesis consistently higher than 10% and a robustness of 100%. All 81 genes were successfully mutated with high efficiencies, 73 of them in the *E. coli* BL21(DE3)Δ*ompA* background, and the remain eight genes, whose inactivation was incompatible with the concomitant presence of *ompA* mutation, were mutated in *E. coli* BL21(DE3).

Considering that the main components of our CRISPR/Cas9 genome editing protocol (pCRISPR-*SacB-gDNA* and pCasRed) are variations-of-the-theme of what already described by others, we believe that our data can be extrapolated to other procedures and therefore could be of general interest. We confirm, thanks to a massive amount of validation data, that CRISPR/Cas genome editing can be very effective and reliable provided that a few experimental solutions are applied. Among those is the use of double stranded synthetic oligonucleotides as donor DNA, and the pre-testing of gDNAs before attempting gene deletion.

Court and co-workers were the first to demonstrate that “λ Red recombineering” in *E. coli* can be carried out with both single and double stranded synthetic oligonucleotides, and that Lg-ss-dDNAs perform better than Ld-ss-dDNAs in promoting the mutagenesis event [31]. Like other authors [10, 18], we confirmed the superiority of the Lg-ss-dDNA even when “λ Red recombineering” is combined with CRISPR/Cas9, a situation whereby the mechanisms that leads to the introduction of the site-specific mutation must be coupled to the mechanisms of double-stranded DNA repair. However, we also found that the successful mutation of all 81 genes could be achieved, with efficiencies consistently higher than 10%, only if ds-dDNA is used (Fig. 3). Furthermore, we showed that ds-dDNA clearly outperformed ss-Lg-dDNA (and ss-Ld-dDNA) when extended deletions were attempted (Table 1).

We did not investigate on the mechanisms of homologous recombination taking place between ds-dDNA and the two chromosomal extremities generated by the Cas9 cleavage. However, it is reasonable to envisage, in addition to other described mechanisms, the contribution of a single exonuclease activity (possibly the 5′ exonuclease provided by the λ Red machinery) acting on both the ds-dDNA and the extremities of the cleaved chromosomal DNA. The exonuclease activity would release single stranded termini, stabilized by Beta, which would become available for base pairing. Such mechanism would not be effective with ss-dDNAs and therefore this could explain the higher efficiency of mutagenesis of ds-dDNA with respect to ss-dDNA. Alternatively, another possible mechanism is that the double strand break is repaired by dsDNA template (and not by ssDNA template) following a Rec-dependent homologous recombination. Such mechanism can occur even in the absence of the lambda red machinery [28, 32]. To test this possibility, the mutagenesis of *proX* and *yiaD*, two genes successfully mutagenized with ds-dDNA only, and with ssDNAs and dsDNA, respectively (Fig. 3), was re-attempted in BL21(DE3)*∆ompA*(pCas9) in which the λ Red machinery was removed. The strain was transformed with either pCRISPR-*SacB* *-proX* or pCRISPR-*SacB* *-yiaD* in the presence of the corresponding ds-dDNAs, and subsequently 100 colonies from each transformation were PCR analyzed. No mutant colonies were identified (data not shown). Therefore, under the experimental conditions we used, the efficiency of dsDNA homologous recombination, if occurring, was at least lower than 1%.

The second factor to consider to achieve high performance is the addition of a rapid experimental step aimed at demonstrating the effectiveness of gDNAs. Theoretically, any sequence in the genome next to the “NGG” (PAM) trinucleotide should represent a potential Cas9 cleavage site. Indeed, most of the gDNAs that we designed and cloned into pCRISPR plasmid produced gRNAs that promoted Cas-mediated cleavage with high specificity. However, 10% of the pCRISPR-*gDNAs* did not work, as judged by their inability to substantially reduce bacterial viability after transformation. We tried to identify the cause root of their failure, such as searching for the presence of sequences forming internal stem-loop structures or partially complementary to other chromosomal regions not carrying the NGG trinucleotides, but we did not find any plausible explanation so far. Whatever reason might be, the use of inefficient gDNAs would affect the overall robustness of the procedure. Therefore, we routinely transform the strain to be mutagenized with all pCRISPR-*gDNAs* before starting mutagenesis. A drop in transformation efficiency of at least three orders of magnitude is indicative of the quality of the gDNAs.

One important aspect to consider in setting up a CRISPR/Cas9-based mutagenesis protocol is the possibility to eliminate the whole machinery once genome editing is terminated. As far as the Cas9/λ Red genes are concerned, since they reside on a low copy number plasmid expressing the Cm^R^, they can be easily removed by growing the strain in the absence of the antibiotic. We experimentally confirmed the effectiveness of losing pCasRed by growing strains overnight in the absence of Cm (see “Methods”). To eliminate the high copy number plasmid encoding the gRNA, a few strategies have been devised, the most popular one being the use of a temperature sensitive origin of replication [10]. To get rid of our pCRISPR-*SacB-gDNA* we envisaged to exploit the toxic effect of the *sacB* gene when *E. coli* is grown in media containing high concentrations of sucrose. *SacB* fused to an antibiotic resistance marker (usually chloramphenicol) has been used in chromosomal gene knock-in/knock-out experiments [21–24] and, more recently, for plasmid curing [25]. Indeed, we found that by simply inoculating the mutant colonies in LB supplemented with 5% sucrose, bacterial cells rapidly lose the plasmid and, if necessary, the culture is ready to be used for an additional round of mutagenesis. Multiple mutations can be introduced in the same recipient strain at a pace of one mutation every two working days (Fig. 5).

The pCRISPR-*SacB-gDNA* plasmid can accommodate more than one gRNA coding sequences potentially allowing the simultaneous modification of more than one gene. We demonstrated that by cloning two gDNAs in the configuration repeat-gDNA_1_-repeat-gDNA_2_-repeat, double gene inactivation was achieved with very high efficiencies. Therefore, not only bacterial cells could successfully take up all three elements simultaneously [pCRISPR-(gDNA)_2_-*SacB* and two dDNAs] but also the gRNA was efficiently transcribed and processed to promote the Cas9-mediated cleavage at both chromosomal sites. It will be interesting to investigate the limits of the system and see how many loci can be simultaneously modified.

## Methods

### Bioinformatics

The “Keio collection” [29], a collection of systematic single-gene knockout mutants of *E. coli*, was used as a basis to evaluate both the biological functions and the non-essentiality of the selected genes listed in Additional file 1: Table S5. To properly select the gDNA and to avoid off-target effects of Cas9, we used the following procedure. First, we identified the PAM sequences in the first half of the target genes. Next, we selected the 30 bp upstream the PAM sequences and we blasted them [33] against *E. coli* BL21(DE3) genome. Those sequences with no complementary nucleotides within the ten nucleotide seed-region and with a homology lower than 15% in the remaining part of the guide were selected.

### Bacterial strains and culture conditions

*Escherichia coli* DH5α strain was routinely grown in LB broth (SIGMA) at 37 °C and used for cloning experiments. *E. coli* BL21(DE3)*∆ompA* strain generated as previously reported [34], was grown in LB broth at 37 or 30 °C when required, and was employed for genome editing experiments. Stock preparations of strains were prepared in LB + 20% glycerol and stored at −80 °C. Each bacterial manipulation was started using an overnight culture from a frozen/glycerol stock. When required, kanamycin and chloramphenicol were added to final concentrations of 50 or 25 μg/ml, respectively.

### Construction of plasmids

Information about all primers, plasmids and *E. coli* strains used in this study are provided in Additional file 1: Tables S1, S5.

The pCasRed plasmid carries a chloramphenicol resistance gene (*Cm* ^*R*^) and encodes the Cas9 nuclease, the λ Red (Exo, Beta, Gam) cassette and the tracrRNA. The *Cas9* gene and the tracrRNA coding sequence are under the control of constitutive promoters while the λ Red gene cassette is transcribed using an arabinose-inducible promoter.

The pCas9 plasmid (ADDGENE #4287 [9]) was used as template for the construction of pCasRed plasmid as follows. The λ Red cassette was PCR amplified from pKOBEG plasmid [20] and was cloned into pCas9 plasmid using the polymerase incomplete primer extension (PIPE) cloning method [35]. Briefly, the vector pCas9 was linearized by V-PIPE PCR amplification with primers Pipe1 pCAS9-F/Pipe1 pCAS9-R. The insert, the λ Red cassette, was I-PIPE PCR amplified with primers redF/redR, which contain 5′ sequences complementary to the two distinct ends of the amplified vector. In this manner, annealing occurred directionally by mixing the PCR products, V-PCR and I-PCR, and by transforming *E. coli* HK-100 strain, pCasRed plasmid was generated. The resulting construct was analyzed by DNA sequencing and used to transform *E. coli* BL21(DE3)*∆ompA* electrocompetent cells.

The pCRISPR-*SacB* plasmid, derived from pCRISPR plasmid (ADDGENE #42875, [9]), where a kanamycin resistance gene (Km^R^) is fused to the *sacB* gene encoding the *Bacillus subtilis* levansucrase, carries the synthetic DNA fragment (gDNA) coding for the guide RNA necessary to drive the Cas9-dependent double stranded break at the desired site of the bacterial genome. The construction of the pCRISPR-*SacB* plasmid was carried out by the PIPE method in two steps. In a first step, the kanamycin resistance cassette of pCRISPR plasmid was replaced by a “cat-sac cassette” containing the chloramphenicol acetyltransferase gene, along with the *sacB* gene, from pKM154 plasmid (ADDGENE #13035) [24]. The vector pCRISPR was linearized by V-PIPE PCR amplification with primers pipeCRISPR-F and pipeCRISPR-R to exclude the kanamycin gene. The insert, the cat-sac cassette, was I-PIPE PCR amplified with cat/sac-pipeF and cat/sac-pipeR primers, which contain 5′ sequence complementary to the two distinct ends of the amplified vector. In this manner, annealing occurred directionally by mixing the PCR products, V-PCR and I-PCR, and after transformation pCRISPR-CatSacB plasmid was isolated. In a second step, the chloramphenicol resistance cassette of the pCRISPR-CatSacB plasmid was then replaced with the kanamycin gene from the original pCRISPR plasmid. The pCRISPR-*CatSacB* plasmid was linearized by using V-crSac F and V-crSac R primers, excluding the chloramphenicol acetyltransferase gene. The kanamycin gene was amplified from pCRISPR plasmid using primers I-kanaF and I-kanaR. The PCR products were then mixed together and used to transform *E. coli*, generating the pCRISPR-*SacB* plasmid. The resulting construct was analyzed by DNA sequencing.

Plasmids expressing the gRNAs, were constructed by phosphorylation and annealing of oligonucleotides (gDNAs) listed in Additional file 1: Table S2, followed by ligation into pCRISPR (or pCRISPR-*SacB*) digested with *BsaI* (New England BioLabs), generating the plasmids listed in Additional file 1: Table S1. The resulting constructs were used to transform *E. coli* DH5α strain (Invitrogen) and the plasmids prepared by QIAprep Spin Miniprep Kit, Qiagen (QIAGEN kit) were analyzed by DNA sequencing.

### Competent cell preparation

For all mutagenesis experiments, *E. coli* BL21(DE3)*∆ompA* carrying pCasRed plasmid was used. To prepare *E. coli* BL21(DE3)*∆ompA*(pCasRed) a 5 ml overnight culture (LB medium) inoculated from a single colony of BL21(DE3)*∆ompA* obtained from an LB-agar plate was grown at 37 °C under vigorous agitation. The overnight culture was diluted 100-fold and grown at 37 °C (200 r.p.m.) until the optical density at 600 nm (OD_600_) reached 0.6–0.8 (~3 h). Then the cells were harvested at 4000 r.p.m. for 20 min at 4 °C and washed three times with cold MilliQ water. After a final wash in 10% glycerol the cells were aliquoted and stored at −80 °C. 50 μl of competent cells were then electroporated using 1 mm Gene Pulser cuvette (Bio-Rad) at 1.8 kV with 1 ng of pCasRed plasmid. Competent cells of *E. coli* BL21(DE3)*∆ompA*(pCasRed) were prepared by growing a single colony in LB medium with 25 μg/ml chloramphenicol at 37 °C under shaking. The overnight culture was diluted to an OD_600_ of 0.1 and grown at 37 °C under shaking (200 r.p.m.) to an OD_600nm_ of 0.2 and then l-arabinose was added to a final concentration of 0.2% for λ Red induction. After induction, the culture was grown to an OD_600_ of 0.7 and then cells were washed and aliquoted as described above.

The transformation efficiency of BL21(DE3)*∆ompA*(pCasRed) electrocompetent cells was of 0.5–2 × 10^6^ CFUs/μg of the “empty” pCRISPR plasmid.

### Gene knockout using CRISPR-Cas9

The genes encoding *ompF, lpp* and *fecA* were used as targets to establish the proof of concept for genome editing via CRISPR-Cas9 system in *E. coli*. All mutagenesis oligonucleotides (donor DNA or dDNAs) (Additional file 1: Table S3) (Sigma-Aldrich) were HPLC purified grade. The dDNAs were designed to delete a region ranging from 30 (∆30) to 2325 (∆2325) nucleotides from target genes, removing the protospacer and PAM regions, thus disrupting the Cas9 cleavage site and at the same time adding an in-frame stop codon downstream the deleted region.

For the leading and lagging strand design, the oligonucleotides annealing to the 3′ > 5′ strand moving clockwise from *OriC* up to *ter* were Ls-ss-dDNAs, while oligonucleotides annealing to the same strand but moving counterclockwise from *OriC* up to *ter* were Lg-ss-dDNAs. The opposite was true for the oligonucleotides annealing to the 5′ > 3′ strand moving clockwise from *OriC* up to *ter* and for those annealing to the same strand moving counterclockwise from *OriC* to *ter*.

The ds-dDNAs were generated by annealing 10 µg of both forward and reverse oligonucleotides in a total volume of 20 µl at 95 °C for 5 min and allowing the reaction mixture to cool down at room temperature. The annealing reaction was verified by loading 500 ng of each single stranded oligonucleotides and 1 μg of total DNA in the annealing reaction and by visualizing the bands using ATLAS ClearSight DNA Stain (BIOATLAS). The bands corresponding to the single stranded oligonucleotides disappeared in the annealing reaction sample. For CRISPR/Cas9-mediated gene knockouts 50 μl of *E. coli* BL21(DE3)*∆ompA*(pCasRed) or BL21(DE3)(pCasRed) competent cells, corresponding to 10^9^ competent cells, were electroporated using 1 mm Gene Pulser cuvette (Bio-rad) at 1.8 kV with 100 ng of pCRISPR-*gDNA* and different quantities of dDNA ranging from 1 to 100 μg. As control, 100 ng of an empty pCRISPR plasmid was used. Cells were then immediately re-suspended in 1 ml of LB medium and allowed to recover at 30 °C for 3 h under agitation before being plated on LB agar with 25 μg/ml chloramphenicol and 50 μg/ml kanamycin and incubated at 37 °C overnight. Mutants were screened by colony PCR using GoTaq master mix (Promega-M7123). Briefly, cells were picked from individual colony using a pipette tip, directly resuspended in PCR reaction mix and DNA amplification was carried out according to the standard cycling GoTaq protocol. The deletion in *ompF* gene was analyzed by using primers: seqs-ompF 1F/ompR (∆30), seqs-ompF 1F/ompR2 (∆100), ompF 1F/ompF 4R2 (∆500, ∆1089) (Additional file 1: Table S5). The deletion of *lpp* was analyzed by using primers: seqs-lpp F/seqs-lpp R (∆30, ∆237) (Additional file 1: Table S5). The deletion in *fecA* gene was analyzed by using primers: FecA_F1/FecA_R1 (∆30), dFecA-seqF/dFecA-seqR (∆500, ∆2325) (Additional file 1: Table S5).

The protocol described above was further validated on 78 genes (Additional file 1: Table S4) using dDNAs (10 μg) designed to delete a region of 30 (∆30 nt) nucleotides (see above) as described above. For simultaneous gene knockout experiments pCRISPR-*ompF_5′G*-*ompF_3′I* and pCRISPR-*lpp_B*-*fecA* *_B* plasmids were constructed by inserting the synthetic sequences into the *BsaI* site (Additional file 1: Table S2). The plasmids (100 ng) were used to co-transform *E. coli* BL21(DE3)*∆ompA*(pCasRed) with dDNA couples (10 μg each donor) ompF_5′G-120∆30/ompF_3′I-120-∆30 and lpp_B-120-∆30/fecA_B-120-∆30, respectively (Additional file 1: Table S3). Transformants were analyzed by colony PCR using primers seqs-ompF 1F/ompR (∆30 at the 5′) and ompF 3′F/ompF 3′R, and seqs-lpp F/seqs-lpp R (∆30) and FecA_F1/FecA_R1 (∆30) (Additional file 1: Table S5).

To test mutagenesis efficiency in the absence of the lambda red machinery, E. coli BL21(DE3)*∆ompA*(pCas9) was transformed with either 100 ng pCRISPR-*SacB*-*proX*/10 µg ds-donor-proX-70-∆30 or 100 ng pCRISPR-*SacB* *-yiaD*/10 μg ds-donor-yiaD-70-∆30 targeting genes *proX* and *yiaD*, respectively. Hundred transformant colonies from each transformation were analyzed by colony PCR using primer s041_proX_F/s041_proX_R and s072_yiaD_F/s072_yiaD_R, respectively.

### Plasmid curing and creation of multiple mutations by stepwise approach

**Fig. 6:**
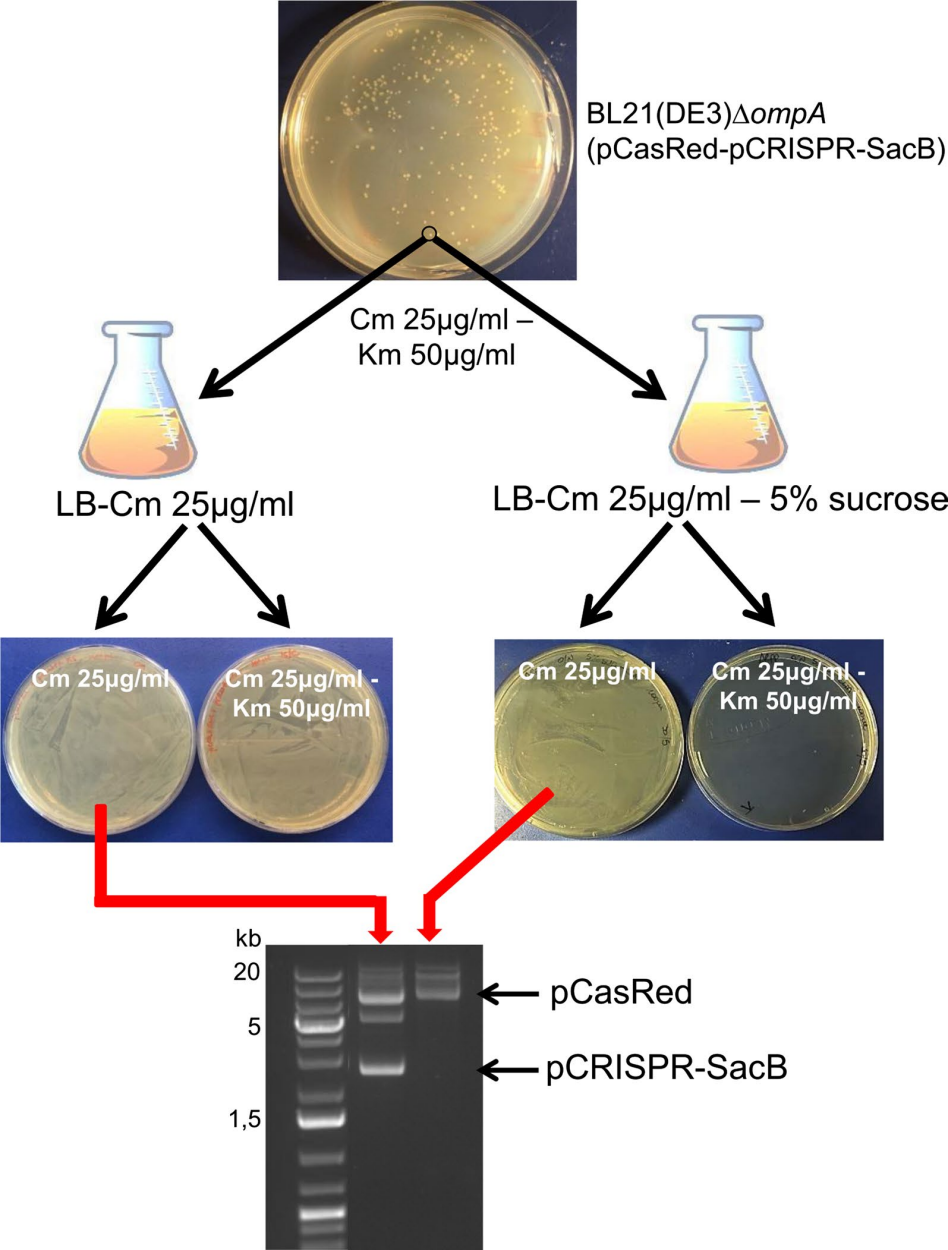
pCRISPR-*SacB-gDNA* plasmid curing using 5% sucrose containing medium. A single colony from *E. coli* BL21(DE3)Δ*ompA* strain carrying both the pCasRed (Cm resistance) and pCRISPR-*SacB-gDNA* (Km resistance) was grown at 37 °C in LB-medium containing 5% sucrose and 25 μg/ml Cm. After 14 h growth, 100 μl of culture were plated on LB-agar plates containing either Cm (25 μg/ml) + Km (50 μg/ml) or Cm (25 μg/ml) alone. The loss of pCRISPR-*SacB-gDNA* plasmid was verified by 1.5% agarose gel analysis of plasmids extracted from bacteria directly collected from the Cm-containing agar plate. As control, the same colony was grown in the absence of 5% sucrose and plasmid extraction was carried out from bacteria collected from LB-agar plate containing 25 μg/ml Cm

To cure mutant strains from pCRISPR plasmid derivatives after each round of mutation, we adopted the strategy proposed by Hale and co-workers [25] by introducing the suicide *sacB* gene downstream of the Km cassette into the pCRISPR plasmid, generating the pCRISPR-*SacB* plasmid. *E. coli* strains carrying any pCRISPR-*SacB* plasmid derivative can survive in the presence of sucrose only if they rapidly lose the plasmid [21–24]. To test the effectiveness of this strategy, we transformed the BL21(DE3)*∆ompA*(pCasRed) strain with pCRISPR-*SacB* and one transformant colony was grown in LB medium supplemented either with 25 μg/ml Cm alone (the antibiotic resistance marker of pCas9-λ Red plasmid) or with 25 μg/ml Cm and 5% sucrose. After overnight growth, 100 μl of each culture were plated on LB agar plates supplemented with either Km and Cm, or Cm only. Not a single colony from the sucrose-containing culture could be isolated on the Km/Cm containing plate (Fig. 6), indicating that all bacteria had lost the pCRISPR-*SacB* plasmid. By contrast, confluent growth was observed on the Km/Cm plate seeded with bacteria grown in sucrose-deprived medium. The loss of pCRISPR plasmid from BL21(DE3)*∆ompA*(pCasRed)(pCRISPR-*SacB*) strain grown in the presence of sucrose was confirmed by plasmid extraction (Fig. 6).

For pCRISPR plasmid curing after each mutation round, the mutant colony, carrying both the pCasRed and pCRISPR-*SacB* plasmids, was inoculated in LB medium containing 5% sucrose and 25 μg/ml chloramphenicol, and grown overnight at 37 °C under shaking conditions. The overnight culture was directly used to prepare competent cells as described above. Briefly, at day 1, 50 μl of *E. coli* BL21(DE3)*∆ompA*(pCasRed) competent cells were co-transformed with 100 ng of pCRISPR*-SacB-fecA_B* plasmid and 10 μg of fecA_B-120-∆30 dDNA. At day 2, transformant colonies were PCR screened and one mutant clone was inoculated in 5 ml LB supplemented with 5% sucrose and 25 μg/ml chloramphenicol. The overnight culture was used to prepare competent cells as described above. The new recipient strain BL21(DE3)*∆ompA/∆fecA*(pCasRed) was co-transformed with 100 ng of pCRISPR-*SacB-lpp_B* plasmid and 10 μg of ds-dDNA lpp_B-120-∆237 and transformant colonies were selected on LB-Agar 50 μg/ml kanamycin and 25 μg/ml chloramphenicol. The day after transformant colonies were PCR screened to identify the *E. coli* BL21(DE3)*∆ompA/∆fecA/∆lpp*(pCasRed) mutant.

The curing of the pCasRed plasmid was carried out after overnight growth at 37 °C under shaking in the absence in the culture medium of chloramphenicol. The day after the culture was plated for single colony on LB-agar plate and colonies were analyzed for chloramphenicol resistance. No chloramphenicol resistance colonies were recovered.

## Additional files

**Additional file 1: Tables S1–S6.** Additional tables.

**Additional file 2: Figure S1.** Additional figure.

## Abbreviations

CRISPR: clustered regularly interspaced short palindromic repeats
PAM: protospacer adjacent motif
CM: chloramphenicol
KM: kanamycin
gDNA: guide DNA
NHEJ: non-homologous end-joining
tracrRNA: trans-activating crRNA
dDNA: donor DNA/synthetic oligonucleotides
Lg-ss-dDNA: single strand donor DNA targeting the lagging strand
Ld-ss-dDNA: single strand donor DNA targeting the leading strand
ds-dDNA: double strand donor DNA
PIPE: polymerase incomplete primer extension

## References

[1] Zhang Y (1998). A new logic for DNA engineering using recombination in *Escherichia coli*. Nat Genet.

[2] Datta S, Costantino N, Court DL (2006). A set of recombineering plasmids for gram-negative bacteria. Gene.

[3] Murphy KC (1998). Use of bacteriophage lambda recombination functions to promote gene replacement in *Escherichia coli*. J Bacteriol.

[4] Muyrers JP (1999). Rapid modification of bacterial artificial chromosomes by ET-recombination. Nucleic Acids Res.

[5] Ellis HM (2001). High efficiency mutagenesis, repair, and engineering of chromosomal DNA using single-stranded oligonucleotides. Proc Natl Acad Sci USA.

[6] Yu D (2000). An efficient recombination system for chromosome engineering in *Escherichia coli*. Proc Natl Acad Sci USA.

[7] Yu D (2003). Recombineering with overlapping single-stranded DNA oligonucleotides: testing a recombination intermediate. Proc Natl Acad Sci USA.

[8] Sharan SK (2009). Recombineering: a homologous recombination-based method of genetic engineering. Nat Protoc.

[9] Jiang W (2013). RNA-guided editing of bacterial genomes using CRISPR-Cas systems. Nat Biotechnol.

[10] Pyne ME (2015). Coupling the CRISPR/Cas9 system with lambda red recombineering enables simplified chromosomal gene replacement in *Escherichia coli*. Appl Environ Microbiol.

[11] Doudna JA, Charpentier E (2014). Genome editing. The new frontier of genome engineering with CRISPR-Cas9. Science.

[12] Sternberg SH, Doudna JA (2015). Expanding the Biologist’s Toolkit with CRISPR-Cas9. Mol Cell.

[13] Singh V, Braddick D, Dhar PK (2017). Exploring the potential of genome editing CRISPR-Cas9 technology. Gene.

[14] Choi KR, Lee SY (2016). CRISPR technologies for bacterial systems: current achievements and future directions. Biotechnol Adv.

[15] Barrangou R, van Pijkeren JP (2016). Exploiting CRISPR-Cas immune systems for genome editing in bacteria. Curr Opin Biotechnol.

[16] Jakociunas T, Jensen MK, Keasling JD (2016). CRISPR/Cas9 advances engineering of microbial cell factories. Metab Eng.

[17] Mougiakos I (2016). Next generation prokaryotic engineering: the CRISPR-Cas toolkit. Trends Biotechnol.

[18] Reisch CR, Prather KL (2015). The no-SCAR (scarless Cas9 assisted recombineering) system for genome editing in *Escherichia coli*. Sci Rep.

[19] Bassalo MC (2016). Rapid and efficient one-step metabolic pathway integration in *E. coli*. ACS Synth Biol.

[20] Derbise A (2003). A rapid and simple method for inactivating chromosomal genes in Yersinia. FEMS Immunol Med Microbiol.

[21] Gay P (1985). Positive selection procedure for entrapment of insertion sequence elements in gram-negative bacteria. J Bacteriol.

[22] Gay P (1983). Cloning structural gene sacB, which codes for exoenzyme levansucrase of *Bacillus subtilis*: expression of the gene in *Escherichia coli*. J Bacteriol.

[23] Steinmetz M (1983). Genetic analysis of sacB, the structural gene of a secreted enzyme, levansucrase of *Bacillus subtilis* Marburg. Mol Gen Genet.

[24] Murphy KC, Campellone KG, Poteete AR (2000). PCR-mediated gene replacement in *Escherichia coli*. Gene.

[25] Hale L (2010). An efficient stress-free strategy to displace stable bacterial plasmids. Biotechniques.

[26] Shuman S, Glickman MS (2007). Bacterial DNA repair by non-homologous end joining. Nat Rev Microbiol.

[27] Wright DG, et al. *Mycobacterium tuberculosis* and *Mycobacterium marinum* non-homologous end-joining proteins can function together to join DNA ends in *Escherichia coli*. Mutagenesis. 2016. 10.1093/mutage/gew042 PMC5989629 27613236

[28] Cui L, Bikard D (2016). Consequences of Cas9 cleavage in the chromosome of *Escherichia coli*. Nucleic Acids Res.

[29] Baba T (2006). Construction of *Escherichia coli* K-12 in-frame, single-gene knockout mutants: the Keio collection. Mol Syst Biol.

[30] Jiang Y (2015). Multigene editing in the *Escherichia coli* genome via the CRISPR-Cas9 system. Appl Environ Microbiol.

[31] Court DL, Sawitzke JA, Thomason LC (2002). Genetic engineering using homologous recombination. Annu Rev Genet.

[32] Meddows TR, Savory AP, Lloyd RG (2004). RecG helicase promotes DNA double-strand break repair. Mol Microbiol.

[33] Altschul SF (1990). Basic local alignment search tool. J Mol Biol.

[34] Fantappie L, et al. Antibody-mediated immunity induced by engineered *Escherichia coli* OMVs carrying heterologous antigens in their lumen. J Extracell Vesicles. 2014;3. 10.3402/jev.v3.24015 PMC4131003 25147647

[35] Klock HE, Lesley SA (2009). The polymerase incomplete primer extension (PIPE) method applied to high-throughput cloning and site-directed mutagenesis. Methods Mol Biol.

