# Supplementary material for: Large scale validation of an efficient CRISPR/Cas-based multi gene editing protocol in Escherichia coli
Source: Microb Cell Fact. 2017 Apr 24;16:68. doi: 10.1186/s12934-017-0681-1 (PMC5404680; doi:10.1186/s12934-017-0681-1)
Supplement: Supplementary file 1 — Additional file 1: Tables S1–S6. Additional tables. [file 12934_2017_681_MOESM1_ESM.pdf]

Supplementary table 1. List of strains and plasmids used throughout our experiments.

Strains and plasmids	**Genotype/relevant characteristics**	**Source**
Strains		
E.coli DH5α	*F- φ80lacZ∆M15 ∆(lacZYA-argF) U169 recA1 endA1 hsdR17 (rk-, mk+) gal- phoA supE44 λ- thi-1 gyrA96 relA1*	Invitrogen
E. coli BL21(DE3)∆*ompA*	*fhuA2 [lon] ompT gal (λ DE3) [dcm] ∆hsdS λ DE3 = λ sBamHIo ∆EcoRI-B int::(lacI::PlacUV5::T7 gene1) i21 ∆nin5-∆**ompA***	Lab stock
E. coli BL21(DE3)∆*ompA/* ∆*ompF*	Derived from *E. coli* BL21(DE3)∆*ompA*- *ompF* knockout	this study
E. coli BL21(DE3)∆*ompA/* ∆*lpp*	Derived from *E. coli* BL21(DE3)∆*ompA*- *lpp* knockout	this study
E. coli BL21(DE3)∆*ompA/* ∆*fecA*	Derived from *E. coli* BL21(DE3)∆*ompA*- *fecA* knockout	this study
E. coli BL21(DE3)∆*ompA/* ∆*lpp/*∆*fecA*	Derived from *E. coli* BL21(DE3)∆*ompA*- *lpp* and *fecA* knockout	this study
Plasmids		
pKOBEG	*E.coli* vector containing the lambda red cassette (pSC101ts, cm, araC-ParaB-gam-bet-exo)	Lab stock
pKM154	*E.coli* vector containing B.subtilis sacB gene (pBR322 ori, Amp, Cm, sacB)	Addgene plasmid # 13036
pCas9	*E.coli* vector containing the S.pyogenes cas9 gene and the tracrRNA (p15A ori, Cm, cas9, tracrRNA)	Addgene plasmid # 42876
pCasRed	*E.coli* vector containing the S.pyogenes cas9 gene and the tracrRNA and the lambda red cassette(p15A ori, Cm, cas9, tracrRNA, araC-ParaB-gam-bet-exo)	this study
pCRISPR-gDNA	*E.coli* vector containing a gRNA expression cassette for Cas9 (pBR322 ori, km, gDNA); guide sequences (Table S2) are introduced into this construct	Addgene plasmid # 42875
pCRISPR-SacB-gDNA	*E.coli* vector containing a gRNA expression cassette for Cas9 and the sacB gene (pBR322 ori, km, gDNA, sacB); guide sequences (Table S2) are introduced into this construct	this study
pCRISPR-ompF_5'A	Derived from pCRISPR to target *E.coli ompF* gene	this study
pCRISPR-ompF_5'B	Derived from pCRISPR to target *E.coli ompF* gene	this study
pCRISPR-ompF_5'C	Derived from pCRISPR to target *E.coli ompF* gene	this study
pCRISPR-ompF_5'D	Derived from pCRISPR to target *E.coli ompF* gene	this study
pCRISPR-ompF_5'E	Derived from pCRISPR to target *E.coli ompF* gene	this study
pCRISPR-ompF_5'F	Derived from pCRISPR to target *E.coli ompF* gene	this study
pCRISPR-ompF_5'G	Derived from pCRISPR to target *E.coli ompF* gene	this study
pCRISPR-ompF_3'H	Derived from pCRISPR to target *E.coli ompF* gene	this study
pCRISPR-ompF_3'I	Derived from pCRISPR to target *E.coli ompF* gene	this study
pCRISPR-ompF_3'L	Derived from pCRISPR to target *E.coli ompF* gene	this study
pCRISPR-ompF_3'M	Derived from pCRISPR to target *E.coli ompF* gene	this study
pCRISPR-ompF_3'N	Derived from pCRISPR to target *E.coli ompF* gene	this study
pCRISPR-lpp_A	Derived from pCRISPR to target *E.coli lpp* gene	this study
pCRISPR-lpp_B	Derived from pCRISPR to target *E.coli lpp* gene	this study
pCRISPR-lpp_C	Derived from pCRISPR to target *E.coli lpp* gene	this study
pCRISPR-lpp_D	Derived from pCRISPR to target *E.coli lpp* gene	this study
pCRISPR-fecA_A	Derived from pCRISPR to target *E.coli fecA*gene	this study
pCRISPR-fecA_B	Derived from pCRISPR to target *E.coli fecA*gene	this study
pCRISPR-fecA_C	Derived from pCRISPR to target *E.coli fecA*gene	this study

pCRISPR-fecA_D	Derived from pCRISPR to target *E.coli fecA*gene	this study
pCRISPR-SacB-fecA_B	Derived from pCRISPR-SacB to target *E.coli fecA*gene	this study
pCRISPR-SacB-lpp_B	Derived from pCRISPR-SacB to target *E.coli lpp*gene	this study
pCRISPR-ompF_5'G-ompF 3'I	Derived from pCRISPR to target *E.coli ompF* gene at the 5' and 3' ends	this study
pCRISPR-lpp_B-fecA_B	Derived from pCRISPR to target *E.coli lpp* and *fecA* genes	this study
pCRISPR-SacB-agp	Derived from pCRISPR-SacB to target *E.coli agp*gene	this study
pCRISPR-SacB-ais	Derived from pCRISPR-SacB to target *E.coli ais* gene	this study
pCRISPR-SacB-artI	Derived from pCRISPR-SacB to target *E.coli artI* gene	this study
pCRISPR-SacB-bglX	Derived from pCRISPR-SacB to target *E.coli bglX* gene	this study
pCRISPR-SacB-cirA	Derived from pCRISPR-SacB to target *E.coli cirA* gene	this study
pCRISPR-SacB-degP	Derived from pCRISPR-SacB to target *E.coli degP* gene	this study
pCRISPR-SacB-degQ	Derived from pCRISPR-SacB to target *E.coli degQ* gene	this study
pCRISPR-SacB-dsbA	Derived from pCRISPR-SacB to target *E.coli dsbA* gene	this study
pCRISPR-SacB-dsbC	Derived from pCRISPR-SacB to target *E.coli dsbC* gene	this study
pCRISPR-SacB-ecnB	Derived from pCRISPR-SacB to target *E.coli ecnB* gene	this study
pCRISPR-SacB-eco	Derived from pCRISPR-SacB to target *E.coli eco* gene	this study
pCRISPR-SacB-fadL	Derived from pCRISPR-SacB to target *E.coli fadL* gene	this study
pCRISPR-SacB-fepA	Derived from pCRISPR-SacB to target *E.coli fepA* gene	this study
pCRISPR-SacB-fhuA	Derived from pCRISPR-SacB to target *E.coli fhuA* gene	this study
pCRISPR-SacB-fkpA	Derived from pCRISPR-SacB to target *E.coli fkpA* gene	this study
pCRISPR-SacB-glnH	Derived from pCRISPR-SacB to target *E.coli glnH* gene	this study
pCRISPR-SacB-glpQ	Derived from pCRISPR-SacB to target *E.coli glpQ* gene	this study
pCRISPR-SacB-gltI	Derived from pCRISPR-SacB to target *E.coli gltI* gene	this study
pCRISPR-SacB-hisJ	Derived from pCRISPR-SacB to target *E.coli hisJ* gene	this study
pCRISPR-SacB-hlpA	Derived from pCRISPR-SacB to target *E.coli hlpA* gene	this study
pCRISPR-SacB-kpsD	Derived from pCRISPR-SacB to target E.coli kpsD gene	this study
pCRISPR-SacB-lamB	Derived from pCRISPR-SacB to target *E.coli lamB* gene	this study
pCRISPR-SacB-malE	Derived from pCRISPR-SacB to target *E.coli malE* gene	this study
pCRISPR-SacB-malM	Derived from pCRISPR-SacB to target *E.coli malM* gene	this study
pCRISPR-SacB-mdoG	Derived from pCRISPR-SacB to target *E.coli mdoG* gene	this study
pCRISPR-SacB-mipA	Derived from pCRISPR-SacB to target *E.coli mipA* gene	this study
pCRISPR-SacB-mltA	Derived from pCRISPR-SacB to target *E.coli mltA* gene	this study
pCRISPR-SacB-mltE	Derived from pCRISPR-SacB to target *E.coli mltE* gene	this study
pCRISPR-SacB-nlpB	Derived from pCRISPR-SacB to target *E.coli nlpB* gene	this study
pCRISPR-SacB-nlpD	Derived from pCRISPR-SacB to target *E.coli nlpD* gene	this study
pCRISPR-SacB-nmpC_1	Derived from pCRISPR-SacB to target *E.coli nmpC_1* gene	this study
pCRISPR-SacB-ompX	Derived from pCRISPR-SacB to target *E.coli ompX* gene	this study
pCRISPR-SacB-oppA	Derived from pCRISPR-SacB to target *E.coli oppA* gene	this study

pCRISPR-SacB-osmE	Derived from pCRISPR-SacB to target *E.coli osmE* gene	this study
pCRISPR-SacB-pal	Derived from pCRISPR-SacB to target *E.coli pal* gene	this study
pCRISPR-SacB-phoE	Derived from pCRISPR-SacB to target *E.coli phoE* gene	this study
pCRISPR-SacB-potD	Derived from pCRISPR-SacB to target *E.coli potD* gene	this study
pCRISPR-SacB-ppiA	Derived from pCRISPR-SacB to target *E.coli ppiA* gene	this study
pCRISPR-SacB-prc	Derived from pCRISPR-SacB to target *E.coli prc* gene	this study
pCRISPR-SacB-proX	Derived from pCRISPR-SacB to target *E.coli proX* gene	this study
pCRISPR-SacB-rcsF	Derived from pCRISPR-SacB to target *E.coli rcsF* gene	this study
pCRISPR-SacB-rlpA	Derived from pCRISPR-SacB to target *E.coli rlpA* gene	this study
pCRISPR-SacB-slp	Derived from pCRISPR-SacB to target *E.coli slp* gene	this study
pCRISPR-SacB-slyB	Derived from pCRISPR-SacB to target *E.coli slyB* gene	this study
pCRISPR-SacB-smpA	Derived from pCRISPR-SacB to target *E.coli smpA* gene	this study
pCRISPR-SacB-surA	Derived from pCRISPR-SacB to target *E.coli surA* gene	this study
pCRISPR-SacB-tolB	Derived from pCRISPR-SacB to target *E.coli tolB* gene	this study
pCRISPR-SacB-tolC	Derived from pCRISPR-SacB to target *E.coli tolC* gene	this study
pCRISPR-SacB-tsx	Derived from pCRISPR-SacB to target *E.coli tsx* gene	this study
pCRISPR-SacB-vacJ	Derived from pCRISPR-SacB to target *E.coli vacJ* gene	this study
pCRISPR-SacB-yajG	Derived from pCRISPR-SacB to target *E.coli yajG* gene	this study
pCRISPR-SacB-ybaY	Derived from pCRISPR-SacB to target *E.coli ybaY* gene	this study
pCRISPR-SacB-ybcU	Derived from pCRISPR-SacB to target *E.coli ybcU* gene	this study
pCRISPR-SacB-ybgF	Derived from pCRISPR-SacB to target *E.coli ybgF* gene	this study
pCRISPR-SacB-ybiS	Derived from pCRISPR-SacB to target *E.coli ybiS* gene	this study
pCRISPR-SacB-ycdO	Derived from pCRISPR-SacB to target *E.coli ycdO* gene	this study
pCRISPR-SacB-yceI	Derived from pCRISPR-SacB to target *E.coli yceI* gene	this study
pCRISPR-SacB-ycfM	Derived from pCRISPR-SacB to target *E.coli ycfM* gene	this study
pCRISPR-SacB-ycfR	Derived from pCRISPR-SacB to target *E.coli ycfR* gene	this study
pCRISPR-SacB-ydcL	Derived from pCRISPR-SacB to target *E.coli ydcL* gene	this study
pCRISPR-SacB-ydgH	Derived from pCRISPR-SacB to target *E.coli ydgH* gene	this study
pCRISPR-SacB-yfeY	Derived from pCRISPR-SacB to target *E.coli yfeY* gene	this study
pCRISPR-SacB-yfgL	Derived from pCRISPR-SacB to target *E.coli yfgL* gene	this study
pCRISPR-SacB-ygdI	Derived from pCRISPR-SacB to target *E.coli ygdI* gene	this study
pCRISPR-SacB-ygdR	Derived from pCRISPR-SacB to target *E.coli ygdR* gene	this study
pCRISPR-SacB-yggE	Derived from pCRISPR-SacB to target *E.coli yggE* gene	this study
pCRISPR-SacB-yggG	Derived from pCRISPR-SacB to target *E.coli yggG* gene	this study
pCRISPR-SacB-ygiW	Derived from pCRISPR-SacB to target *E.coli ygiW* gene	this study
pCRISPR-SacB-yhcN	Derived from pCRISPR-SacB to target *E.coli yhcN* gene	this study
pCRISPR-SacB-yhjJ	Derived from pCRISPR-SacB to target *E.coli yhjJ* gene	this study
pCRISPR-SacB-yiaD	Derived from pCRISPR-SacB to target *E.coli yiaD* gene	this study

pCRISPR-SacB-yifL	Derived from pCRISPR-SacB to target *E.coli yifL* gene	this study
pCRISPR-SacB-yjeI	Derived from pCRISPR-SacB to target *E.coli yjeI* gene	this study
pCRISPR-SacB-yncD	Derived from pCRISPR-SacB to target *E.coli yncD* gene	this study
pCRISPR-SacB-yncE	Derived from pCRISPR-SacB to target *E.coli yncE* gene	this study
pCRISPR-SacB-yraM	Derived from pCRISPR-SacB to target *E.coli yraM* gene	this study
pCRISPR-SacB-yraP	Derived from pCRISPR-SacB to target *E.coli yraP* gene	this study
pCRISPR-SacB-yrbC	Derived from pCRISPR-SacB to target *E.coli yrbC* gene	this study

Supplementary table 2. List of all guide sequences that have been cloned into the pCRISPR-gDNA and/or pCRISPR-SacB-gDNA plasmid (Table S1). Asterisks designate guides that did not work as judged by transformation efficiency (see text).

gDNA name	***Sequences forward (upper line) and reverse (lower line)***
ompF 5'A	aaacggcagtgatcgtccctgctctgttagtagcg
	aaaacgctactaacagagcagggacgatcactgcc
ompF 5'B	aaaccaacaaagtagatctgtacggtaaagctgtg
	aaaacacagctttaccgtacagatctactttgttg
ompF 5'C	aaacgtaaagctgtcggtctgcattatttctccag
	aaaactggagaaataatgcagaccgacagctttac
ompF 5'D	aaactaaagctgtcggtctgcattatttctccaag
	aaaacttggagaaataatgcagaccgacagcttta
ompF 5'E	aaactgtcggtctgcattatttctccaagggtaag
	aaaacttacccttggagaaataatgcagaccgaca
ompF 5'F	aaacctccaagggtaacggtgaaaacagttacggg
	aaaacccgtaactgttttcaccgttacccttggag
ompF 5'G	aaacgacatgacctatgcccgtcttggttttaaag
	aaaactttaaaaccaagacgggcataggtcatgtc
ompF 3'H	aaaccatcaaccagatcgattctgacaacaaactg
	aaaacagtttgttgtcagaatcgatctggttgatg
ompF 3'I	aaaccaaatctaaagcgaaagacgtagaaggtatg
	aaaacataccttctacgtctttcgctttagatttg
ompF 3'L	aaacttacaccaaatctaaagcgaaagacgtagag
	aaaactctacgtctttcgctttagatttggtgtaa
ompF 3'M	aaactctgttagttgcgcaataccagttcgatttg
	aaaacaaatcgaactggtattgcgcaactaacaga
ompF 3'N	aaaccaacggtaaaaaagctgaacagtgggctacg
	aaaacgtagcccactgttcagcttttttaccgttg
lpp A	aaactactaaactggtactgggcgcggtaatcctg
	aaaacaggattaccgcgcccagtaccagtttagta
lpp B	aaactgggcgcggtaatcctgggttctactctgcg
	aaaacgcagagtagaacccaggattaccgcgccca
lpp C	aaacacgacgtgaacgcaatgcgttccgacgttcg
	aaaacgaacgtcggaacgcattgcgttcacgtcgt
lpp D	aaacctggagcaacctgccagcagagtagaacccg
	aaaacgggttctactctgctggcaggttgctccag
fecA A	aaaccgttacgcgtttttcgtaaaacaacaccttg
	aaaacaaggtgttgttttacgaaaaacgcgtaacg
fecA B	aaacagtatgccgcacacagcggatttaccctctg
	aaaacagagggtaaatccgctgtgtgcggcatact
fecA C	aaaccagcaggctcaggcgaatggcgttaaccaag
	aaaacttggttaacgccattcgcctgagcctgctg
fecA D	aaacagcaaaagcggaaaacgagagaccggccagg
	aaaacctggccggtctctcgttttccgcttttgct

ompF_5'G-ompF 3'I	aaacGACATGACCTATGCCCGTCTTGGTTTTAAAgttttagagctatgctgttttgaatggtcccaaaacCAAATCTAAAGCGAAAGACGTAGAAGGTATg
	aaaacATACCTTCTACGTCTTTCGCTTTAGATTTGgttttgggaccattcaaaacagcatagctctaaaacTTTAAAACCAAGACGGGCATAGGTCATGTC
lpp_B-fecA_B	aaacTGGGCGCGGTAATCCTGGGTTCTACTCTGCgttttagagctatgctgttttgaatggtcccaaaacAGTATGCCGCACACAGCGGATTTACCCTCTg
	aaaacAGAGGGTAAATCCGCTGTGTGCGGCATACTgttttgggaccattcaaaacagcatagctctaaaacGCAGAGTAGAACCCAGGATTACCGCGCCCA
agp-G-1r	aaacccattgttcgccagcggcgcacgtaagttag
	aaaactaacttacgtgcgccgctggcgaacaatgg
ais-G-1f	aaacatggtttgccacgtatcgacaataaaacacg
	aaaacgtgttttattgtcgatacgtggcaaaccat
artI-G-1f *	aaacacaaccagatcgttggttttgacgtcgaccg
	aaaacggtcgacgtcaaaaccaacgatctggttgt
artI-G-2r	aaaccaatcgattcaaacggaggataggaggcttg
	aaaacaagcctcctatcctccgtttgaatcgattg
bglX-G-1f	aaacttggtcagctgcgcttaatcagcgtaggccg
	aaaacggcctacgctgattaagcgcagctgaccaa
cirA-G-2r	aaactggcaggtgcatctttaagattttgttccag
	aaaactggaacaaaatcttaaagatgcacctgcca
degP-G-1f	aaaccactgagtgcactggctctgagtttaggttg
	aaaacaacctaaactcagagccagtgcactcagtg
degQ-G-1f	aaaccattagcgttaagtgtcgggttaactctctg
	aaaacagagagttaacccgacacttaacgctaatg
dsbA-G-1f *	aaacgtttagcgcatcggcggcgcagtatgaagag
	aaaactcttcatactgcgccgccgatgcgctaaac
dsbA-G-2r	aaacgcagaagaaagagaaaaactccagcacttgg
	aaaaccaagtgctggagtttttctctttcttctgc
dsbC-G-1f	aaaccgcggcaattcaacaaacgttagccaaaatg
	aaaacattttggctaacgtttgttgaattgccgcg
ecnB-G-1f	aaactaaataacattacctaaaaggaagacgttag
	aaaactaacgtcttccttttaggtaatgttattta
eco-G-1f *	aaactgaatctaccctgaaagtagaactgttaatg
	aaaacattaacagttctactttcagggtagattca
eco-G-2r	aaacacgcttcatcccttttcagcttgtggatag
	aaaactatccacaagctgaaaaagggatgaagcgt
fadL-G-2r	aaacattgcgccttcccctgaataagcccggcccg
	aaaacgggccgggcttattcaggggaaggcgcaat
fepA-G-1f	aaactaccgccgccgagcagaacttacaggcgccg
	aaaacggcgcctgtaagttctgctcggcggcggta
fhuA-G-1f	aaacaatcgcagttgtagtagccacagcggttagg
	aaaacctaaccgctgtggctactacaactgcgatt
fkpA-G-1f	aaacaaatgacgatcagaaatcagcttatgcactg
	aaaacagtgcataagctgatttctgatcgtcattt
glnH-G-1f	aaacataaatatgtgggctttgacgttgatctgtg
	aaaacacagatcaacgtcaaagcccacatatttat
glpQ-G-1f	aaaccatggcgatcatgatgagcactatagtcatg
	aaaacatgactatagtgctcatcatgatcgccatg

gltI-G-1f	aaaccaatcctggccctggcgctttccgcaggacg
	aaaacgtcctgcggaaagcgccagggccaggattg
hisJ-G-1f	aaacgtttgctgcgattccgcaaaacatccgcatg
	aaaacatgcggatgttttgcggaatcgcagcaaac
hlpA-G-1f	aaacggttattagctgcaggtctcggtttagcacg
	aaaacgtgctaaaccgagacctgcagctaataacc
kpsD-G-1f	aaaccgggcaacagtcggatacgcaaaacatgagg
	aaaacctcatgttttgcgtatccgactgttgcccg
lamB-G-1f	aaaccggcggtgaacaacagtgtttccagactacg
	aaaacgtagtctggaaacactgttgttcaccgccg
malE-G-1f	aaacaatctggattaacggcgataaaggctataag
	aaaacttatagcctttatcgccgttaatccagatt
malM-G-1f	aaacctgctgcgctgcaacaactcacctggacacg
	aaaacgtgtccaggtgagttgttgcagcgcagcag
mdoG-G-1f	aaacgatgtcgcaaagcaagctcaatctttagctg
	aaaacagctaaagattgagcttgctttgcgacatc
mipA-G-1f	aaaccgataacttctggttccgtggcttaggtggg
	aaaacccacctaagccacggaaccagaagttatcg
mltA-G-1f	aaacaagacgggaaatttacccagcctttctctcg
	aaaacgagagaaaggctgggtaaatttcccgtctt
mltE-G-1f	aaacattgatcacggcgattatcgctatcgaatcg
	aaaacgattcgatagcgataatcgccgtgatcaat
nlpB-G-1f	aaacaagcggcaccgcttgcggagcttcatgcccg
	aaaacgggcatgaagctccgcaagcggtgccgctt
nlpD-G-1f	aaactaatggcaatgcgcctgcaaatactaattcg
	aaaacgaattagtatttgcaggcgcattgccatta
nmpC_1-G-1f	aaactaataaagacagtaacaagctggatctgtag
	aaaactacagatccagcttgttactgtctttatta
ompX-G-1f	aaactacttccgtagctgcgacttctactgtaacg
	aaaacgttacagtagaagtcgcagctacggaagta
oppA-G-1f	aaactggcagctgatgtacccgcaggcgtcacacg
	aaaacgtgtgacgcctgcgggtacatcagctgcca
osmE-G-1f	aaaccggcggtattaaccatgctggcgggttgtag
	aaaactacaacccgccagcatggttaataccgccg
pal-G-1f	aaactgaaagggctgatgattgctctgcctgttag
	aaaactaacaggcagagcaatcatcagccctttca
phoE-G-1f	aaactaacaaagacggtaataaactggatgtctag
	aaaactagacatccagtttattaccgtctttgtta
potD-G-1f	aaacggtcacgccacctgctcgcggcgggtgctcg
	aaaacgagcacccgccgcgagcaggtggcgtgacc
ppiA-G-1f	aaacggacccgcacgtattgttgacaacctcagcg
	aaaacgctgaggttgtcaacaatacgtgcgggtcc
prc-G-1f	aaacgttctcattatcgccagttcgacctcgatcg
	aaaacgatcgaggtcgaactggcgataatgagaac
proX-G-1f	aaacgctgctggtcagtcgtgcgctggagaaattg
	aaaacaatttctccagcgcacgactgaccagcagc

rcsF-G-1f	aaacaccgatctgtttagtagcactcatgctaagg
	aaaaccttagcatgagtgctactaaacagatcggt
rlpA-G-1f	aaacggtaagtgtaccgcagcctgcggtatgtaag
	aaaacttacataccgcaggctgcggtacacttacc
slp-G-1f	aaactgctgttcataaccagccggggttatatgtg
	aaaacacatataaccccggctggttatgaacagca
slyB-G-1f	aaacgattcagggcggtgatgattccaacgttatg
	aaaacataacgttggaatcatcaccgccctgaatc
smpA-G-1f	aaacgcaaacgctgacgctgacctttaacagtagg
	aaaacctactgttaaaggtcagcgtcagcgtttgc
surA-G-1f	aaactgatcgcgaataccagtttcgctgccccccg
	aaaacggggggcagcgaaactggtattcgcgatca
tolB-G-1f	aaaccgacagcggtgtagattccggtcgtcctatg
	aaaacataggacgaccggaatctacaccgctgtcg
tolC-G-1f	aaacgcgcagtccattactgccacagctaggtttg
	aaaacaaacctagctgtggcagtaatggactgcgc
tsx-G-1f	aaacgaaaacgacaaaccgcagtatctttccgacg
	aaaacgtcggaaagatactgcggtttgtcgttttc
vacJ-G-1f	aaacagatcagcaagggcgttctgacccgttagag
	aaaactctaacgggtcagaacgcccttgctgatct
yajG-G-1f	aaacttacactgccacagcaggatccaagcctgag
	aaaactcaggcttggatcctgctgtggcagtgtaa
ybaY-G-1f	aaacacaacctgctatccagcaaccgaatgtctcg
	aaaacgagacattcggttgctggatagcaggttgt
ybcU-G-1f	aaaccccatcacttcttcgtttcgggaattggacg
	aaaacgtccaattcccgaaacgaagaagtgatggg
ybgF-G-1f	aaactgctcaggcaccaatcagtagtgtcggctcg
	aaaacgagccgacactactgattggtgcctgagca
ybiS-G-1f	aaaccttatcctctgccaaccgacgggagtcgccg
	aaaacggcgactcccgtcggttggcagaggataag
ycdO-G-1f	aaaccggataagcagtgcgaaccgatgaccattag
	aaaactaatggtcatcggttcgcactgcttatccg
yceI-G-1f	aaaccatccagcaccttggctatagctggttatag
	aaaactataaccagctatagccaaggtgctggatg
ycfM-G-1f	aaacgcgctggcgatgtttctcgccgggtgtgtgg
	aaaaccacacacccggcgagaaacatcgccagcgc
ycfR-G-1f *	aaactacaatcagtgctaacgcggggacaaatctg
	aaaacagatttgtccccgcgttagcactgattgta
ycfR-G-2r	aaacgcgttgactgaacttcgaccgccgcaaagcg
	aaaacgctttgcggcggtcgaagttcagtcaacgc
ydcL-G-1f *	aaacttgcgaaagttgcagctttatgcggcttatg
	aaaacataagccgcataaagctgcaactttcgcaa
ydcL-G-2r	aaaccccaacgtaaaacaggtttacctgtagccgg
	aaaaccggctacaggtaaacctgttttacgttggg
ydgH-G-1f *	aaacccctcctggcgtcggcactgctttctgctag
	aaaactagcagaaagcagtgccgacgccaggaggg

ydgH-G-2f	aaaccggcagcggttaaaccttttgaccgtgtagg
	aaaacctacacggtcaaaaggtttaaccgctgccg
yfeY-G-1f	aaactcgtcagttaactggtctgccgctaacccgg
	aaaaccgggttagcggcagaccagttaactgacga
yfgL-G-1f	aaacgaccacggcgtggagcacttccgttggtagg
	aaaacctaccaacggaagtgctccacgccgtggtc
ygdI-G-1f	aaaccaccaatgacggacgtaccatcgtctctgag
	aaaactcagagacgatggtacgtccgtcattggtg
ygdR-G-1f	aaacgaccaaagatggccgtatgattttgaccgag
	aaaactcggtcaaaatcatacggccatctttggtc
yggE-G-1f	aaacccctggcggcattaatgggtattagcgggag
	aaaactcccgctaatacccattaatgccgccaggg
yggG-G-1f	aaacaagccctgagcgatcaggcatgtcaggagag
	aaaactctcctgacatgcctgatcgctcagggctt
ygiW-G-1f	aaacggcaacgcaaagtcaggccggaggattccag
	aaaactggaatcctccggcctgactttgcgttgcc
yhcN-G-1f	aaactcttgaagcaatcgggaccgtatctgtaagg
	aaaaccttacagatacggtcccgattgcttcaaga
yhjJ-G-2r	aaacggcgaatttcaacacgatcgctgggacgctg
	aaaacagcgtcccagcgatcgtgttgaaattcgcc
yiaD-G-1f	aaacctgctatcggcgcaggtctgggctctctcgg
	aaaaccgagagagcccagacctgcgccgatagcag
yifL-G-1f	aaacattacttactctcttcagcctgacgggctgg
	aaaaccagcccgtcaggctgaagagagtaagtaat
yjeI-G-1f	aaacgcaactggctttccgggcaacacggagaagg
	aaaaccttctccgtgttgcccggaaagccagttgc
yncD-G-1f	aaacccgagctggatacgccagcagcagtaagcgg
	aaaaccgcttactgctgctggcgtatccagctcgg
yncE-G-1f	aaacttcatcgcgcctgcgtggttcattactgttg
	aaaacaacagtaatgaaccacgcaggcgcgatgaa
yraM-G-1f	aaacccgatcagtccactgcttatatgcagggtag
	aaaactaccctgcatataagcagtggactgatcgg
yraP-G-1f *	aaacgtgtcggcacccaggtggacgatggtacccg
	aaaacgggtaccatcgtccacctgggtgccgacac
yraP-G-2f	aaacaagaagcgcgcattaatgtaacggcctatcg
	aaaacgataggccgttacattaatgcgcgcttctt
yrbC-G-1f	aaactcgctttgctggtgattgcacctctgagtgg
	aaaaccactcagaggtgcaatcaccagcaaagcga

Supplementary table 3. Donor DNAs.

Donor DNA ID	***Sequence (5'-3')***
ompF 5'G-120-Δ30	gtcggtctgcattatttctccaagggtaacggtgaaaacagttacggtggcaatggctaagaaactcaaatcaattccgatctgaccggttatggtcagtgggaatataacttccagggt
ompF 5'G-120-Δ30 R	accctggaagttatattcccactgaccataaccggtcagatcggaattgatttgagtttcttagccattgccaccgtaactgttttcaccgttacccttggagaaataatgcagaccgac
ompF 5'G-70-Δ30	agggtaacggtgaaaacagttacggtggcaatggcagggtaactaagaaaacagttacggtggcaatggc
ompF 5'G-70-Δ100	gcaatggcgacatgacctatgcccgtcttggttaaaacaaaacgcgtctggcattcgcgggtcttaaata
ompF 5'G-120-Δ100	ggtaacggtgaaaacagttacggtggcaatggcgacatgacctatgcccgtcttggttaaaacaaaacgcgtctggcattcgcgggtcttaaatacgctgacgttggttctttcgattac
ompF 5'G-120-Δ100 R	gtaatcgaaagaaccaacgtcagcgtatttaagacccgcgaatgccagacgcgttttgttttaaccaagacgggcataggtcatgtcgccattgccaccgtaactgttttcaccgttacc
ompF 5'G-70-Δ500	gcgacatgacctatgcccgtcttggttttaaataaggctactggtctgaagtacgacgcgaacaacatct
ompF 5'G-120-Δ500	ggtgaaaacagttacggtggcaatggcgacatgacctatgcccgtcttggttttaaataaggctactggtctgaagtacgacgcgaacaacatctacctggcagcgaactacggtgaaac
ompF 5'G-120-Δ500 R	gtttcaccgtagttcgctgccaggtagatgttgttcgcgtcgtacttcagaccagtagccttatttaaaaccaagacgggcataggtcatgtcgccattgccaccgtaactgttttcacc
ompF 3'I-120-Δ30	gttctgttagttgcgcaataccagttcgatttcggtctgcgtccgtccatcgcttacacctaagatgttgatctggtgaactactttgaagtgggtgcaacctactacttcaacaaaaac
ompF 3'I-120-Δ30 R	gtttttgttgaagtagtaggttgcacccacttcaaagtagttcaccagatcaacatcttaggtgtaagcgatggacggacgcagaccgaaatcgaactggtattgcgcaactaacagaac
ompF 5'G-120-Δ1089	gacagaacttattgacggcagtggcaggtgtcataaaaaaaaccatgagggtaataaatatagcacacctctttgttaaatgccgaaaaaacaggactttggtcctgtttttttatacc
ompF 5'G-120-Δ1089 R	ggtataaaaaaacaggaccaaagtcctgtttttcggcatttaacaaagaggtgtgctatatttattaccctcatggttttttttatgacacctgccactgccgtcaataagttctgtc
fecA B-70-Δ30	atcagtatgccgcacacagcggatttaccctctagtttgacgccagcctgacgcgcggcaagcagagcaa
fecA B-120-Δ30	tttccgcttttgctgcacaggttaatatcgcaccgggatcgctcgataaagcgctcaattaattgacgccagcctgacgcgcggcaagcagagcaacggcctgcacggcgattacgacgt
fecA B-120-Δ30 R	acgtcgtaatcgccgtgcaggccgttgctctgcttgccgcgcgtcaggctggcgtcaattaattgagcgctttatcgagcgatcccggtgcgatattaacctgtgcagcaaaagcggaaa
fecA B-120-Δ100	ccgggatcgctcgataaagcgctcaatcagtatgccgcacacagcggatttaccctctaataataaaaccgctgggaaataacagctggacgctggagcccgcgcccgcaccaaaagaag
fecA B-120-Δ100 R	cttcttttggtgcgggcgcgggctccagcgtccagctgttatttcccagcggttttattattagagggtaaatccgctgtgtgcggcatactgattgagcgctttatcgagcgatcccgg

fecA B-120-Δ500	ccgggatcgctcgataaagcgctcaatcagtatgccgcacacagcggatttaccctctaataaggtacgcggtggtggtgcggtgcg ttacggaccgcagagcgtgggcggcgtggtgaa
fecA B-120-Δ500 R	ttcaccacgccgcccacgctctgcggtccgtaacgcaccgcaccaccaccgcgtaccttattagagggtaaatccgctgtgtgcggc atactgattgagcgctttatcgagcgatcccgg
fecA B-120-Δ2325	ctttttacccttctcgttcgactcatagctgaacacaacaaaaatgatgatggggaaggttgtgttaacgcccggcttgccgggcttttag ctggaatgtgattatgttggcatttatcc
fecA B-120-Δ2325 R	ggataaatgccaacataatcacattccagctaaaagcccggcaagccgggcgttaacacaaccttccccatcatcatttttgttgtgtt cagctatgagtcgaacgagaagggtaaaaag
lpp B-70-Δ30	agagggtattaataatgaaagctactaaactggtataagcaggttgctccagcaacgctaaaatcgatca
lpp B-70-Δ30 R	tgatcgattttagcgttgctggagcaacctgcttataccagtttagtagctttcattattaataccctct
lpp B-120-Δ30	cgctacatggagattaactcaatctagagggtattaataatgaaagctactaaactggtataagcaggttgctccagcaacgctaaa atcgatcagctgtcttctgacgttcagactctg
lpp B-120-Δ30 R	cagagtctgaacgtcagaagacagctgatcgattttagcgttgctggagcaacctgcttataccagtttagtagctttcattattaatacc ctctagattgagttaatctccatgtagcg
lpp B-120-Δ237	aactttgtgtaatacttgtaacgctacatggagattaactcaatctagagggtattaatatagtacctgtgaagtgaaaaatggcgcaca ttgtgcgccatttttttgtctgccgttta
lpp B-120-Δ237 R	taaacggcagacaaaaaaaatggcgcacaatgtgcgccatttttcacttcacaggtactatattaataccctctagattgagttaatctc catgtagcgttacaagtattacacaaagtt
agp-70-Δ30	tatcagctacagcaagtgctcatgatgagctaaagtgtgctggagcagtcgacgccgaataaatggccag
agp-70-Δ30 R	ctggccatttattcggcgtcgactgctccagcacactTTAgctcatcatgagcacttgctgtagctgata
ais-70-Δ30	ggactgggtactcatgccgcctggagtagctaagccagactggcacagcagcacccggttgtcgttttgt
ais-70-Δ30 R	acaaaacgacaaccgggtgctgctgtgccagtctggcttagctactccaggcggcatgagtacccagtcc
artI-70-Δ30	acagctgccgaaaccattcgttttgctacctaagcaaacaaccagatcgttggttttgacgtcgacctgg
artI-70-Δ30 R	ccaggtcgacgtcaaaaccaacgatctggttgtttgcttaggtagcaaaacgaatggtttcggcagctgt
bglX-70-Δ30	ctgcttaagaaaatgacagttgatgagaaataagataatccgaaagaggcgatccgcgagatgatcaaag
bglX-70-Δ30 R	ctttgatcatctcgcggatcgcctctttcggattatcttatttctcatcaactgtcattttcttaagcag
cirA-70-Δ30	ggcgaaacgatggttgtcactgcatcttcctaaatcagcgtcattacccaggaagacctgcagcgaaaac
cirA-70-Δ30 R	gttttcgctgcaggtcttcctgggtaatgacgctgatttaggaagatgcagtgacaaccatcgtttcgcc
degP-70-Δ30	agactgaaatacatgaaaaaaaccacattataagcgttatctccgctctctgcaacggcggctgagac
degP-70-Δ30 R	gtctcagccgccgttgcagagagcggagataacgcttataatgtggttttttcatgtatttcagtct
degQ-70-Δ30	ataatgaaaaaacaaacccagctgttgagttaagcgtcatttcaggccgtcgcgtcgattccaggccagg

degQ-70-Δ30 R	cctggcctggaatcgacgcgacggcctgaaatgacgcttaactcaacagctgggtttgtttttcattat
dsbA-70-Δ30	actaccctggaaaaaccggtagctggcgcgtaacactgctatcagtttgaagaagttctgcatatttctg
dsbA-70-Δ30 R	cagaaatatgcagaacttcttcaaactgatagcagtgttacgcgccagctaccggtttttccagggtagt
dsbC-70-Δ30	gcggcgttttcaggttttgctcaggctgattaaatcaaaagcagcgatattcagcccgcgcccgtagctg
dsbC-70-Δ30 R	cagctacgggcgcgggctgaatatcgctgcttttgatttaatcagcctgagcaaaacctgaaaacgccgc
ecnB-70-Δ30	ctataggcaaacataaataacattacctaataagcgatcttttctgttctggtgctttcaacagtattaa
ecnB-70-Δ30 R	ttaatactgttgaaagcaccagaacagaaaagatcgcttattaggtaatgttatttatgtttgcctatag
eco-70-Δ30	gaaagcgtccagccactggaaaaaatcgcgtaacaggtgattcagttaaccccgcaagaagatgaatcta
eco-70-Δ30 R	tagattcatcttcttgcggggttaactgaatcacctgttacgcgatttttttccagtggctggacgctttc
fadL-70-Δ30	attgaggttatggtcatgagccagaaaacctaagcacttatctccacccaggcctggtcggcaggctttc
fadL-70-Δ30 R	gaaagcctgccgaccaggcctgggtggagataagtgcttaggttttctggctcatgaccataacctcaat
fepA-70-Δ30	actcctgtttcacatgacgatactattgtctaagtttcgaccatcaccgcagatgaaatccgcaaaaacc
fepA-70-Δ30 R	ggtttttgcggatttcatctgcggtgatggtcgaaacttagacaatagtatcgtcatgtgaaacaggagt
fhuA-70-Δ30	aaaactgctcagccaaaacactcactgcgttaatctgtttatgcacaggcagcggttgaaccgaaagaag
fhuA-70-Δ30 R	cttctttcggttcaaccgctgcctgtgcataaacagattaacgcagtgagtgttttggctgagcagtttt
fkpA-70-Δ30	gctacaactgctgacagcaaagcagcgttctaagcttcgctgggtcgttacatggaaaactctcttaaag
fkpA-70-Δ30 R	ctttaagagagttttccatgtaacgacccagcgaagcttagaacgctgctttgctgtcagcagttgtagc
glnH-70-Δ30	gccttcgttccgtttgaatttaaacagggctaagctgccatcgctaaagagctgaagctggattacgaac
glnH-70-Δ30 R	gttcgtaatccagcttcagctctttagcgatggcagcttagccctgtttaaattcaaacggaacgaaggc
glpQ-70-Δ30	aatggcatgaaattgacgctgaaaaacctttaaagcagtgcaatggcggcggacagcaacgaaaaaaatag
glpQ-70-Δ30 R	ctattttttcgttgctgtccgccgccattgcactgctTTAaaggtttttcagcgtcaatttcatgccatt
gltI-70-Δ30	ttggatatgcaattacgtaaacctgccatataacaggcagatgacgccgccccggcagcgggcagtacgc
hisJ-70-Δ30	ctggttctggccttctccagcgcaactgcgtaaaccgacccgacctatgcgccatttgaatcaaaaaatt
hisJ-70-Δ30 R	aatttttgattcaaatggcgcataggtcgggtcggtttacgcagttgcgctggagaaggccagaaccag
hlpA-70-Δ30	tgggatggtaaggagtttattgtgaaaaagtaaacttctgctcaggcggctgacaaaattgcaatcgtca
kpsD-70-Δ30	acaggagccgcgccgcttaccggtattctgtaattcgacaataccccgccgcccgcaccgccggtggtca
lamB-70-Δ30	tatgcacgttccggtattggttggacaggttaagctcaaagtaaataccgtcttggcaacgaatgtgaaa

lamB-70-Δ30 R	tttcacattcgttgccaagacggtatttactttgagcttaacctgtccaaccaataccggaacgtgcata
malE-70-Δ30	gctctcgccaaaatcgaagaaggtaaactgtaactcgctgaagtcggtaagaaattcgagaaagataccg
malM-70-Δ30	caaaacaccagcgacgcgccagccattccataagatcaatctaaaacccagaccacccaactggcgaccg
malM-70-Δ30 R	cggtcgccagttgggtggtctgggttttagattgatcttatggaatggctggcgcgtcgctggtgttttg
mdoG-70-Δ30	acatcttcaagctgggctttcagtattgattaaaaaggctacgagacgcccaaaagcaacttgccctccg
mipA-70-Δ30	gtttacccagtaccggtaatcaactatgaataatactacctgtggaatgacgcaacggataaactttcaa
mipA-70-Δ30 R	ttgaaagtttatccgttgcgtcattccacaggtagtattattcatagttgattaccggtactgggtaaac
mltA-70-Δ30	tccaaaccaaccgatcgcggacagcaatattaaaaccagccagatgccgttggcgcgccgattaacgccg
mltA-70-Δ30 R	cggcgttaatcggcgcgccaacggcatctggctggttttaatattgctgtccgcgatcggttggtttgga
mltE-70-Δ30	aaagccggtgcagcctggggcgtcgatccataaggtaatccgaacgcggtgagtaaatcgaatgccattg
mltE-70-Δ30 R	caatggcattcgatttactcaccgcgttcggattaccTTAtggatcgacgccccaggctgcaccggcttt
nlpB-70-Δ30	cgtcaggtcagtggtgatgaagcctacctgtaaggaatgattttgccggtgacctccggtgattatgcaa
nlpB-70-Δ30 R	ttgcataatcaccggaggtcaccggcaaaatcattccTTAcaggtaggcttcatcaccactgacctgacg
nlpD-70-Δ30	acttcaaatccaccggcaccggtcagctcctaattgattacgccgccgccgaaaatggggacgacgtcta
nlpD-70-Δ30 R	tagacgtcgtccccattttcggcggcggcgtaatcaattaggagctgaccggtgccggtggatttgaagt
nmpC_1-70-Δ30	atggcgatgtctgctcaggcagctgaaatttaaaaagttaatgccaagcactacttttcctctaacgatg
nmpC_1-70-Δ30 R	catcgttagaggaaaagtagtgcttggcattaactttttaaatttcagctgcctgagcagacatcgccat
ompX-70-Δ30	gcactggccgcagttctggctttcaccgcataaggttacgcacagagcgacgctcagggccaaatgaaca
ompX-70-Δ30 R	tgttcatttggccctgagcgtcgctctgtgcgtaaccttatgcggtgaaagccagaactgcggccagtgc
oppA-70-Δ30	ctggctgcgctaatggcagggaatgtcgcgtaagaaaaacaaacactggtacgtaacaatggttcagaag
oppA-70-Δ30 R	cttctgaaccattgttacgtaccagtgtttgtttttcttacgcgacattccctgccattagcgcagccag
osmE-70-Δ30	aacaagaatatggcaggaattctgagtgcataatatgatcgtaccaaagaccagtttgtacagcctgtgg
osmE-70-Δ30 R	ccacaggctgtacaaactggtctttggtacgatcatattatgcactcagaattcctgccatattcttgtt
pal-70-Δ30	ggaatcattgaaatgcaactgaacaaagtgtaaattgcggcatgttcttccaacaagaacgccagcaatg
pal-70-Δ30 R	cattgctggcgttcttgttggaagaacatgccgcaatttacactttgttcagttgcatttcaatgattcc
phoE-70-Δ30	gcatctgcatccgtacaggccgcagaaatataaaaagttaaagccatgcattatatgagtgataacgaca
potD-70-Δ30	aggtaacacaggggacgttaaatgaaaaaataactgggcatgagcgccgctcacgccgatgacaacaaca

potD-70-Δ30 R	tgttgttgtcatcggcgtgagcggcgctcatgcccagttattttttcatttaacgtcccctgtgttacct
ppiA-70-Δ30	gctctttctcccgcagcaatggcagcgaaataaaacatcgaactggagctggataaacaaaaagcgccag
ppiA-70-Δ30 R	ctggcgctttttgtttatccagctccagttcgatgttttatttcgctgccattgctgcgggagaaagagc
prc-70-Δ30	gtaagtgagcgcgtaacgtcgcgcttcacctaattttcggccaaaatctttgaccgctacctgaatctgc
prc-70-Δ30 R	gcagattcaggtagcggtcaaagattttggccgaaaattaggtgaagcgcgacgttacgcgctcacttac
proX-70-Δ30	cagagcaccatcactgaagaaaccttccagtaatataccgtcaacaaacccagcgaagtagattacaacg
proX-70-Δ30 R	cgttgtaatctacttcgctgggtttgttgacggtatattactggaaggtttcttcagtgatggtgctctg
rcsF-70-Δ30	atcatacattgaggaaatactatgcgtgcttaatgttccatgttaagcagatcccctgtcgaacccgttc
rlpA-70-Δ30	gcatgtacaagcgatgatggtcagcaacagtaacccatagttgaaattagcggggcggacccgcgtttcg
slp-70-Δ30	aataaccaacctgatattcaaaaaagtttttaacaacaagcgcgctttggtgggaaggttatcaacgtta
slp-70-Δ30 R	taacgttgataaccttcccaccaaagcgcgcttgttgttaaaaactttttgaatatcaggttggttatt
slyB-70-Δ30	atggcaccatcgttaacgtacgtccggtacagtaagcaattggcggtgctgttcttggtggtttcctggg
slyB-70-Δ30 R	cccaggaaaccaccaagaacagcaccgccaattgcttactgtaccggacgtacgttaacgatggtgccat
smpA-70-Δ30	tccgccagcaaccaggtcatgaaggtgtaactcagtaagtgttgaccaatattgataacaaacctgcgct
surA-70-Δ30	tgaagaactggaaaacgctgcttctcggtatcgcctaagtagtcgataaagtcgcagccgtcgtcaataa
tolB-70-Δ30	catcagttctgcatgctgaagtccgcattgtgatctaagttgttcctttccagtgggcggggcctggtgc
tolB-70-Δ30 R	gcaccaggccccgcccactggaaaggaacaacttagatcacaatgcggacttcagcatgcagaactgatg
tolC-70-Δ30	atcgtgatgctgcctttgaaaaaattaatgaagcgtaagcagattacacctatagcaacggctaccgcga
tsx-70-Δ30	cgctctcttcgtcttttactgtcaacgcagcttaatggcaccagagcgttaacgttgtcggaagctatca
tsx-70-Δ30 R	tgatagcttccgacaacgttaacgctctggtgccattaagctgcgttgacagtaaaagacgaagagagcg
vacJ-70-Δ30	cgcttctggtggggtgtgcgagttccggtacataattcaaccgcaccatgtacaacttcaacttcaatgt
vacJ-70-Δ30 R	acattgaagttgaagttgtacatggtgcggttgaattatgtaccggaactcgcacaccccaccagaagcg
yajG-70-Δ30	caaaaccgccaacaactattgaagtttccccgacgtaaggcatcaccgtaagcattaatggtgccgatca
yajG-70-Δ30 R	tgatcggcaccattaatgcttacggtgatgccttacgtcggggaaacttcaatagttgttggcggttttg
ybaY-70-Δ30	cggctgcaaatacgtctatttcagcaacacaataaaccgtctggatccggcagaaagtcgcactgccgcc
ybaY-70-Δ30 R	ggcggcagtgcgactttctgccggatccagacggtttattgtgttgctgaaatagacgtatttgcagccg
ybcU-70-Δ30	acaaaccgacagcagtaacaccaaaggaaaccatctaagagaaaactgttgatgcagccaaaatttgtgg

ybgF-70-Δ30	tactggttggtatagcggccccctgggccgctttttaatcggtcgaagaccgcgtcactcaacttgagcg
ybgF-70-Δ30 R	cgctcaagttgagtgacgcggtcttcgaccgattaaaaagcggcccagggggccgctataccaaccagta
ybiS-70-Δ30	ctgttgtcggcttttgcagtaccgcctctgcggtataagttggtcagaatcaggtgatcaccattcctga
ycdO-70-Δ30	acgccgctgatgtgccgcaggtcaaagtgaccgtgtaagttaacgccgggaaaacacagttcattattca
yceI-70-Δ30	acaaagaaggtcagcacgcctttgttaatttccgctaaacctttaaagatttcgacggtacttttacctt
ycfM-70-Δ30	tgacaaaaatgagtcgctacgccttgattacctaacaacgtgaacctgcaccggtagaagaagtgaaacc
ycfR-70-Δ30	gctgcggcgattttaagctccatgtcattttaagaaggccaacaaaaagtcggtacaatcagtgctaacg
ycfR-70-Δ30 R	cgttagcactgattgtaccgactttttgttggccttcttaaaatgacatggagcttaaaatcgccgcagc
ydcL-70-Δ30	ttaaacaattactctgatttaaaagaaacataagacccgagttttgatcaaagcaaatatgacagcatcg
ydcL-70-Δ30 R	cgatgctgtcatatttgctttgatcaaaactcgggtcttatgtttcttttaaatcagagtaattgtttaa
ydgH-70-Δ30	aacgcagcaacagaactgacaccggagcaataaggtcgttttaatgctattggcgaagcggtgaaagccg
ydgH-70-Δ30 R	cggctttcaccgcttcgccaatagcattaaaacgaccttattgctccggtgtcagttctgttgctgcgtt
yfeY-70-Δ30	ctatgccgttgatgctgaccggctgttccacgatgtaaaactggtttgggtcgtccaccaaagtgagcga
yfeY-70-Δ30 R	tcgctcactttggtggacgacccaaaccagttttacatcgtggaacagccggtcagcatcaacggcatag
yfgL-70-Δ30	ccccattgccaaccgttgaaaaccagtttacgccgtaaattggcaacttctattccaatcttcatccggc
yfgL-70-Δ30 R	gccggatgaagattggaatagaagttgccaatttacggcgtaaactggttttcaacggttggcaatgggg
ygdI-70-Δ30	agcgcctgttccggttcgaactatgtgatgtaaaaaccacagactgataacgataccggtatgatttcgt
ygdI-70-Δ30 R	acgaaatcataccggtatcgttatcagtctgtggtttttacatcacatagttcgaaccggaacaggcgct
ygdR-70-Δ30	ctgtttccgggtgttccagtgattacgtcatggcgtaaaaacctgaaattgatgatgataccgggctggt
yggE-70-Δ30	aacacggagagactaacgtgaagttcaaagttatctaagcagcgcaggctaacgaattgccggatggacc
yggG-70-Δ30	aggcttacagtttgagtgatgcgcaggtgaaataaagcaaggcgacgattgcgccagccaatagcgaata
ygiW-70-Δ30	gcagcagagcagggcggtttttctggcccataaccgaacggcagcgtaacgactgtagaaagcgcaaaat
yhcN-70-Δ30	ctgccgactccattgatgctgcacaagcacaaaattaagtggcgtcttcgccaatggatatgcgtgaaat
yhjJ-70-Δ30	aacggtttacagtggcaagtgctgaccacctaactggttaataccggttcgctcgccgaaagtacacaac
yhjJ-70-Δ30 R	gttgtgtactttcggcgagcgaaccggtattaaccagttaggtggtcagcacttgccactgtaaaccgtt
yiaD-70-Δ30	ctaacccttacaccggcgaacgcgaagcaggtaaataaggcgcgggtattggtgcgctatcttcttcgaa
yifL-70-Δ30	taatgaaaaacgtgtttaaggcactcactgtataactgaaaggtccgctctatttcccgcctgcagataa

yifL-70-Δ30 R	ttatctgcaggcgggaaatagagcggacctttcagttatacagtgagtgccttaaacacgtttttcatta
yjeI-70-Δ30	agtgccagctgattggtactgcgacaggtaagcaataaggcggttctatgcgcggcgcagcaaacgatct
yncD-70-Δ30	aaaccatgattgtcagtgccgcaccgcaggtggtttaagtggatggcgaggagatgcgcctggcaacacc
yncD-70-Δ30 R	ggtgttgccaggcgcatctcctcgccatccacttaaaccacctgcggtgcggcactgacaatcatggttt
yncE-70-Δ30	agggagtcgtcatgcatttacgtcatctgttttaatcattgcttgttgcttcatcattcagtacgcaggc
yncE-70-Δ30 R	gcctgcgtactgaatgatgaagcaacaagcaatgattaaaacagatgacgtaaatgcatgacgactccct
yraM-70-Δ30	cagccctgattttcgccggttgtggcacccatacttaagcgcaggctgattctgccttttatcttcagca
yraM-70-Δ30 R	tgctgaagataaaaggcagaatcagcctgcgcttaagtatgggtgccacaaccggcgaaaatcagggctg
yraP-70-Δ30	agcgcattgtcgaaagacgaacagattaagtaagtgctgctggttgggcagtcaccaaatgctgaacttt
yrbC-70-Δ30	aggagaaccgacgcatgtttaaacgtttaatgatgtaagcaaccgcggcagaccagaccaatccgtataa
yrbC-70-Δ30 R	ttatacggattggtctggtctgccgcggttgcttacatcattaaacgtttaaacatgcgtcggttctcct

Supplementary table 4. List of target genes.

NCBI Accession number	*Gene name*	*Annotation (according to Keio collection)*
YP_002998847.1	agp	periplasmic glucose-1-phosphatase/inositol phosphotase; EC: 3.1.3.10
YP_002999917.1	ais	hypothetical protein
YP_002998710.1	artI	arginine transporter subunit
YP_002999805.1	bglX	periplasmic beta-D-glucoside glucohydrolase; EC:3.2.1.21
YP_002999826.1	cirA	ferric iron-catecholate outer membrane transporter
YP_002998025.2	degP	serine endoprotease (protease Do), membrane-associated; EC: 3.4.21.107
YP_003000803.1	degQ	periplasmic serine endoprotease; EC: 3.4.21.-
YP_003001429.1	dsbA	periplasmic protein disulfide isomerase I; EC:5.3.4.1
YP_003000455.1	dsbC	protein disulfide isomerase II; EC:5.3.4.1
YP_003001706.1	ecnB	entericidin B membrane lipoprotein
YP_002999876.1	eco	ecotin precursor, serin protease inhibitor
YP_003000008.1	fadL	long-chain fatty acid outer membrane transporter; EC:3.1.1.35
YP_003001839.1	fecA	ferric citrate outer membrane transporter; Fe(3+) dicitrate transport protein
YP_002998390.1	fepA	iron-enterobactin outer membrane transporter
YP_002998014.1	fhuA	ferrichrome outer membrane transporter
YP_003000904.1	fkpA	FKBP-type peptidyl-prolyl cis-trans isomerase (rotamase); EC:5.2.1.8
YP_002998634.1	glnH	glutamine ABC transporter periplasmic protein
YP_002999904.1	glpQ	periplasmic glycerophosphodiester phosphodiesterase; EC:3.1.4.46
YP_002998459.1	gltI	glutamate and aspartate transporter subunit
YP_002999973.1	hisJ	histidine/lysine/arginine/ornithine transporter subunit
YP_002998040.1	hlpA	periplasmic chaperone
YP_003000530.1	kpsD	KpsD protein
YP_003001597.1	lamB	maltoporin precursor
YP_002999435.1	lpp	murein lipoprotein
YP_003001595.1	malE	maltose ABC transporter periplasmic protein
YP_003001598.1	malM	maltose regulon periplasmic protein
YP_002998885.1	mdoG	glucan biosynthesis protein, periplasmic
YP_002999534.1	mipA	scaffolding protein for murein synthesizing machinery; MipA family protein
YP_006094274.1	mltA	membrane-bound lytic murein transglycosylase A; EC:3.2.1.-
YP_002999004.1	mltE	lytic murein endotransglycosylase E; EC:2.4.99.-
YP_003000108.1	nlpB	lipoprotein
YP_003000328.1	nlpD	predicted outer membrane lipoprotein
YP_006094171.1	nmpC_1	pseudogene
YP_002998775.1	ompF	outer membrane porin 1a (Ia;b;F)
YP_002998637.1	ompX	outer membrane protein X
YP_002999051.1	oppA	oligopeptide transporter subunit
YP_002999491.1	osmE	DNA-binding transcriptional activator; osmotically inducible lipoprotein
YP_002998534.1	pal	peptidoglycan-associated outer membrane lipoprotein
YP_002998104.1	phoE	outer membrane phosphoporin protein E
YP_002998956.1	potD	spermidine/putrescine ABC transporter periplasmic substrate binding protein
YP_003000921.1	ppiA	peptidyl-prolyl cis-trans isomerase A (rotamase A); EC:5.2.1.8
YP_002999582.1	prc	carboxy-terminal protease for penicillin-binding protein 3; EC:3.4.21.-
YP_003000272.1	proX	glycine betaine transporter subunit

YP_002998059.1	rcsF	predicted outer membrane protein, signal
YP_002998440.1	rlpA	rare lipoprotein A
YP_003001057.1	slp	outer membrane lipoprotein
YP_002999403.1	slyB	outer membrane lipoprotein
YP_003000242.1	smpA	small membrane lipoprotein assembly factor BamE
YP_002997923.1	surA	peptidyl-prolyl cis-trans isomerase (PPIase); EC:5.2.1.8
YP_002998533.1	tolB	translocation protein TolB precursor
YP_003000618.1	tolC	outer membrane channel precursor protein
YP_002998220.1	tsx	nucleoside channel, receptor of phage T6 and colicin K
YP_003000011.1	vacJ	predicted lipoprotein
YP_002998246.1	yajG	predicted lipoprotein
YP_002998266.1	ybaY	predicted outer membrane lipoprotein
YP_002998369.1	ybcU	predicted lipoprotein
YP_002998535.1	ybgF	hypothetical protein
YP_002998642.1	ybiS	hypothetical protein; L,D-transpeptidase YbiS
YP_002998860.1	ycdO	hypothetical protein; iron uptake system component EfeO
YP_002998891.1	yceI	hypothetical protein
YP_002998939.1	ycfM	predicted outer membrane lipoprotein; penicillin-binding protein activator
YP_002998945.1	ycfR	hypothetical protein; multiple stress resistance protein BhsA
YP_002999215.1	ydcL	predicted lipoprotein
YP_002999366.1	ydgH	hypothetical protein
YP_003000071.1	yfeY	hypothetical protein
YP_003000141.1	yfgL	protein assembly complex; lipoprotein component; outer membrane protein assembly factor BamB
YP_003000392.1	ygdI	hypothetical protein
YP_003000412.1	ygdR	hypothetical protein
YP_003000483.1	yggE	hypothetical protein
YP_006094285.1	yggG	predicted peptidase; putative metalloprotease; EC:3.4.24.-
YP_003000607.1	ygiW	hypothetical protein
YP_006094294.1	yhcN	hypothetical protein
YP_003001076.1	yhjJ	predicted zinc-dependent peptidase
YP_003001100.1	yiaD	predicted outer membrane lipoprotein
YP_003001369.1	yifL	predicted lipoprotein
YP_003001701.1	yjeI	hypothetical protein
YP_002999235.1	yncD	predicted iron outer membrane transporter; iron complex outermembrane recepter protein
YP_002999236.1	yncE	hypothetical protein
YP_003000723.1	yraM	hypothetical protein
YP_003000726.1	yraP	hypothetical protein
YP_003000766.1	yrbC	predicted ABC-type organic solvent transporter; phospholipid transport system substrate-binding protein

Supplementary table 5. List of primers.

ID primer	*Primer sequence*
s001_agp_F	cgcaactgtggcagggatagt
s001_agp_R	cacttcgagcacgccaccttt
s002_ais_F	actggcgctcgctgcaattgc
s002_ais_R	actggcgctcgctgcaattgc
s003_artI_F	tctttccgccacagctgccga
s003_artI_R	acggcttctacgcgacggaatt
s004_bglX_F	taggaatcgcggtgagtctggc
s004_bglX_R	gccccaacctgaccgtctttg
s005_cirA_F	gggctgtgtttgtccgctatttc
s005_cirA_R	cgtcagttgtacgccaggcac
s006_degP_F	gcgttatctgttaatcgagactg
s006_degP_R	ccttctacgttaatgctgaccac
s007_degQ_F	tcattcaggtacgagagcagg
s007_degQ_R	ttccttccacccgtacgctca
s008_dsbA_F	ggtaaacagtacactaccctgg
s008_dsbA_R	tgagtcagatctttgcccagg
s009_dsbC_F	ctttgttagcggcgttttcagg
s009_dsbC_R	agccgtgccgctaacgtcata
s010_ecnB_F	tctcccgcgctgccagctaat
s010_ecnB_R	cacgcgtggtgttgcaggcag
s011_eco_F	acctgcagtattgtttgccgc
s011_eco_R	ttcccgccgagacgatgcaa
s012_fadL_F	cctacacttcgcgctcctgtt
s012_fadL_R	tgcgccttcccctgaataagc
s013_fepA_F	cattccctggccttgttggtc
s013_fepA_R	tggcatggtacggatgatctc
s014_fhuA_F	gcgcgttccaaaactgctcag
s014_fhuA_R	gtgccggtagctgactgtcg
s015_fkpA_F	acaatggccgttgccctgcat
s015_fkpA_R	tcctgaacaccagcgatcagc
s016_glnH_F	tgcggtttcttctcatgccgc
s016_glnH_R	gcgccagatcgacgtttttgg
s017_glpQ_F	ctgcaaaaacgcaacggaggc
s017_glpQ_R	agataatccgctccctgcgca
s018_gltI_F	ctcacaacgggtatccatgcg
s018_gltI_R	ctgaagattcacggtgaccgac
s019_hisJ_F	ctggtgctatcgctctctctg
s019_hisJ_R	cgcatccagcggattttcgac
s020_hlpA_F	aggcgatcaatataagatcgccg
s020_hlpA_R	accggttttctgcgctacctg
s021_kpsD_F	tactgattgccgcctgtcacg
s021_kpsD_R	cgctggtgccgttgaaaagttg
s022_lamB_F	tgtctgctcaggcaatggctg

s022_lamB_R	ggccacgttagtgtcgaaatag
s023_malE_F	cgcatcctcgcattatccgca
s023_malE_R	gccgcaacctgtgggaatttc
s024_malM_F	agcgcgcctggaattagcctt
s024_malM_R	agttcgccaatgtttgccggg
s025_mdoG_F	tgcgttggttgagtgctgcag
s025_mdoG_R	gtcttcagattgttccagtacgc
s026_mipA_F	cgtagcgcacgctgaaggtaa
s026_mipA_R	gtaaagcggcgaccagtaagc
s027_mltA_F	gaaaggacgttgggtaaagtacc
s027_mltA_R	cagacgcggtgacgaattacg
s028_mltE_F	catgactatacgaacccgccg
s028_mltE_R	cacgtccggaggttgaagctt
s029_nlpB_F	ctggcaaaggttgcgggtgtt
s029_nlpB_R	atgtccagcgccttaccgaca
s030_nlpD_F	gcccaaaattcaccgttcgcc
s030_nlpD_R	ggctgctgtaccggctgaatt
s031_nmpC_1_F	ggcaatttctgctgtagctgca
s031_nmpC_1_R	gaccgaaaccagtcagttgatc
s032_ompX_F	gcatgtctttcagcactggcc
s032_ompX_R	gcagtacggcttttctcggtg
s033_oppA_F	agagaagtttagtagcagctggc
s033_oppA_R	tcgctgaccagtaagccttcaaa
s034_osmE_F	gaacaagaatatggcaggaattctg
s034_osmE_R	caggatgtaggtctggcaagta
s035_pal_F	caggtcaaattccctgcctgg
s035_pal_R	ttcgcatccataccagtgccg
s036_phoE_F	aagagcactctggcattagtggt
s036_phoE_R	ccataaccagtcagttgatcgttaa
s037_potD_F	tgatggttattgccagccagctt
s037_potD_R	ccggtttctttggtgaactgttc
s038_ppiA_F	gatggctgctgttttcgctcttt
s038_ppiA_R	aagccaggaatgacgcggtga
s039_prc_F	cgcgtgctgatcaaattccgg
s039_prc_R	tcgcgaactgttcaacatcgctt
s040_proX_F	acttttgctgccgatctgccg
s040_proX_R	ttcacggcggtgaaggttgca
s041_rcsF_F	aatatcattcaggacgggcgctt
s041_rcsF_R	ttcggttttgcaggctccgct
s042_rlpA_F	atctgcatcgcggcaggaatg
s042_rlpA_R	cgagacggatcctgcacgatt
s043_slp_F	ggtgcactcatcctcagcctt
s043_slp_R	cagcgatttctaacaacgtatccg
s044_slyB_F	gtcggttgtgttaataacgacacc
s044_slyB_R	gttccgccaccaacagtattcc
s045_smpA_F	tggcatgacgcaacaacaagttg

s045_smpA_R	gttaccactcagcgcaggtttg
s046_surA_F	ccacgtaatccgcagtgcgg
s046_surA_R	gttgctgccttgcctgagcag
s047_tolB_F	ctcatactgtgggcatcagttc
s047_tolB_R	cggctgctgtggcagacga
s048_tolC_F	cagcaagcacgccttagtaac
s048_tolC_R	ggattgagttaactgcagggac
s049_tsx_F	acattactggcagccggtgc
s049_tsx_R	cataaccatagaagtcgaaccag
s050_vacJ_F	agcttcgcctgtcggcgctt
s050_vacJ_R	caaaccgttacgcgccggttg
s051_yajG_F	cgttagttgctctgtttatgcttg
s051_yajG_R	ggaggcggtcagggtaacg
s052_ybaY_F	gttggcggcttgcgcagataa
s052_ybaY_R	tgacggtgcatcggctaacg
s053_ybcU_F	ctgccgctctggcaatgctta
s053_ybcU_R	ccgagcaatccatttacgaatgt
s054_ybgF_F	gtcgtgcggtactggtttact
s054_ybgF_R	tcagaaagttgttgctggagttg
s055_ybiS_F	tctcaacccaatggcctgcca
s055_ybiS_R	ctccagcggctgagtgttac
s056_ycdO_F	cattaacttccgccgtaacgca
s056_ycdO_R	cactccagcgccttctggct
s057_yceI_F	tcgcgtccctgatgttctctg
s057_yceI_R	gtgattagtatcgacgctggtg
s058_ycfM_F	gcgcacaaagtcagactttatct
s058_ycfM_R	cgggatcgtcggcaccgag
s059_ycfR_F	aacgtaaaaaccctcatcgctgc
s059_ycfR_R	gtaatacggaaagattttgcgccc
s060_ydcL_F	cggcttattggctctgtctgg
s060_ydcL_R	cgacggtttcggtaccggata
s061_ydgH_F	agcttaagaacaccctcctgg
s061_ydgH_R	gtcgacaacataaaaagaggcgg
s062_yfeY_F	tgcactccaagcaacgttattga
s062_yfeY_R	tgcagtggtgtggacgccgt
s063_yfgL_F	tactgctgccaggactgctttc
s063_yfgL_R	gtccgctgcatagacaacgttgt
s064_ygdI_F	gactgccgcaattatttctgcct
s064_ygdI_R	ctgatccagttcgaccatctctt
s065_ygdR_F	aacagactattatcataggtgagcc
s065_ygdR_R	cttgctgatcgtgataactcacc
s066_yggE_F	aagcttgcctccagaggtcct
s066_yggE_R	gcaagagtggcaatgtctggc
s067_yggG_F	tggcaacggtactgaccggtt
s067_yggG_R	tgttgcctagcgcattggcaata
s068_ygiW_F	taatcgcagtaatggccctgtg

s068_ygiW_R	gaacacgtagagatcgtcagaga
s069_yhcN_F	ccactgttgctgcattaagcgta
s069_yhcN_R	caccgctacgagcttcagtaat
s070_yhjJ_F	gcggtttgctgatgatggcca
s070_yhjJ_R	gcgtgactgtaaccgctctgt
s071_yiaD_F	agtggtgctctggcggtatct
s071_yiaD_R	atgtaataacccacgccgccg
s072_yifL_F	cgccttctcctgcgatgatag
s072_yifL_R	ccgtggattgcgtttgcgtct
s073_yjeI_F	caacgaattgagtgctgccgg
s073_yjeI_R	ccataaatcacgttaccgcccatt
s074_yncD_F	tccgtccgacagaccgttttg
s074_yncD_R	aaaccaggcacgctggtcagt
s075_yncE_F	caagagcgtaacgatgattacgc
s075_yncE_R	cgtaggcacctttacctaccg
s076_yraM_F	cgtttgaaagccgcgcgttgt
s076_yraM_R	cccggttttaccttctttcacca
s077_yraP_F	tgggtaccaaagccgcaactg
s077_yraP_R	ttggcaccgtctacgcccata
s078_yrbC_F	cagctgctgcgccaggtaataa
s078_yrbC_R	gcggttgctcattcttcaggc
Seqs-ompF 1F	atgatgaagcgcaatattctg
ompR	gatcggaattgatttgagtttc
ompR2	gaatgccagacgcgttttgtt
ompF-1F	gccataacgatcatcctgttac
ompF-4R2	ccgtgcgagcacgtttgtcattg
crispr 2f	gctgagacaaatagtgcgat
NeoR	gcccagtcatagccgaatag
ompF 3'F	cctatgttgactacatcatcaaccagatcg
ompF 3'R	cgatctggttgatgatgtagtcaacatagg
FecA_F1	ctcgttcgactcatagctgaacacaac
FecA_R1	cgtccagcagttgttgcaggcc
FecA_F2	gccattcgcctgagcctgctg
FecA_F1	ctcgttcgactcatagctgaacacaac
FecA R3	ctctgcggtccgtaacgcaccg
dFecA-seqF	caccactgtaaggaaaataattc
dFecA-seqR	gccaacataatcacattccag
seqs-lpp F	gcgttcgatgcttctttgagcg
seqs-lpp R	acgcgtgacgcagtagcggtaaac
Pipe1 pCAS9-F	ctaacggattcaccactccaagaattggagccaatcaattc
Pipe1 pCAS9-R	cgaggtgccgccggcttccattcaggtcgaggtggcccgg
pCas-red1F	cttcgatcattggaccgctg
pCas-red2F	ccattatgattcttctcgct
pCas-red1R	tgcgcattcacagttctccg
pCas-red2R	acaggagcacgatcatgcgc

redF	gccggcggcacctcgcatcgatttattatgacaacttg
redR	tggtgaatccgttagtcatcgccattgctccccaa
pipeCRISPR-F	tgagcgggactctggggttcgagagctcgcttggactcctgttg
pipeCRISPR-R	tggcaattccgacgtctaagaaaccattattatcatgacattaacc
cat\sac-pipeF	acgtcggaattgccataagggcaccaataactgcc
cat\sac-pipeR	ccagagtcccgctcaatcggcattttcttttgcg
I-kanaF	actgccttaaaaaaaaattgaacaagatggattgcacgc
I-kanaR	gctaaggaagctaaacgccttcttgacgagttcttc
V-crSac F	tttagcttccttagctcctgaaaatctcg
V-crSac R	tttttttaaggcagttattggtgccc

Supplementary table6. 30 bp deletion of nine genes in BL21(DE3) wild type using 10µg of ds-dDNA.

**Target gene-pCRISPR-gDNA**	**dDNA ID**	**Efficiency (%)- positive/total**
pCRISPR-gDNA pCRISPR-lamB	lamB-70-Δ30 ds	10% (1/10)
pal pCRISPR-pal	pal-70-Δ30 ds	100% (5/5)
tolB pCRISPR-tolB	tolB-70-Δ30 ds	33% (2/6)
yfeY pCRISPR-yfeY	yfeY-70-Δ30 ds	40% (4/10)
yhjJ pCRISPR-yhjJ	yhjJ-70-Δ30 ds	10% (1/10)
yifL pCRISPR-yifL	yifL-70-Δ30 ds	33% (1/3)
nmpC pCRISPR-nmpC	nmpC-70-Δ30 ds	10% (1/10)
yajG pCRISPR-yajG	yajG-70-Δ30 ds	50% (1/2)
