# Supplementary material for: Large scale validation of an efficient CRISPR/Cas-based multi gene editing protocol in Escherichia coli
Source: Microb Cell Fact. 2017 Apr 24;16:68. doi: 10.1186/s12934-017-0681-1 (PMC5404680; doi:10.1186/s12934-017-0681-1)
Supplement: Supplementary file 2 — Additional file 2: Figure S1. Additional figure. [file 12934_2017_681_MOESM2_ESM.pdf]

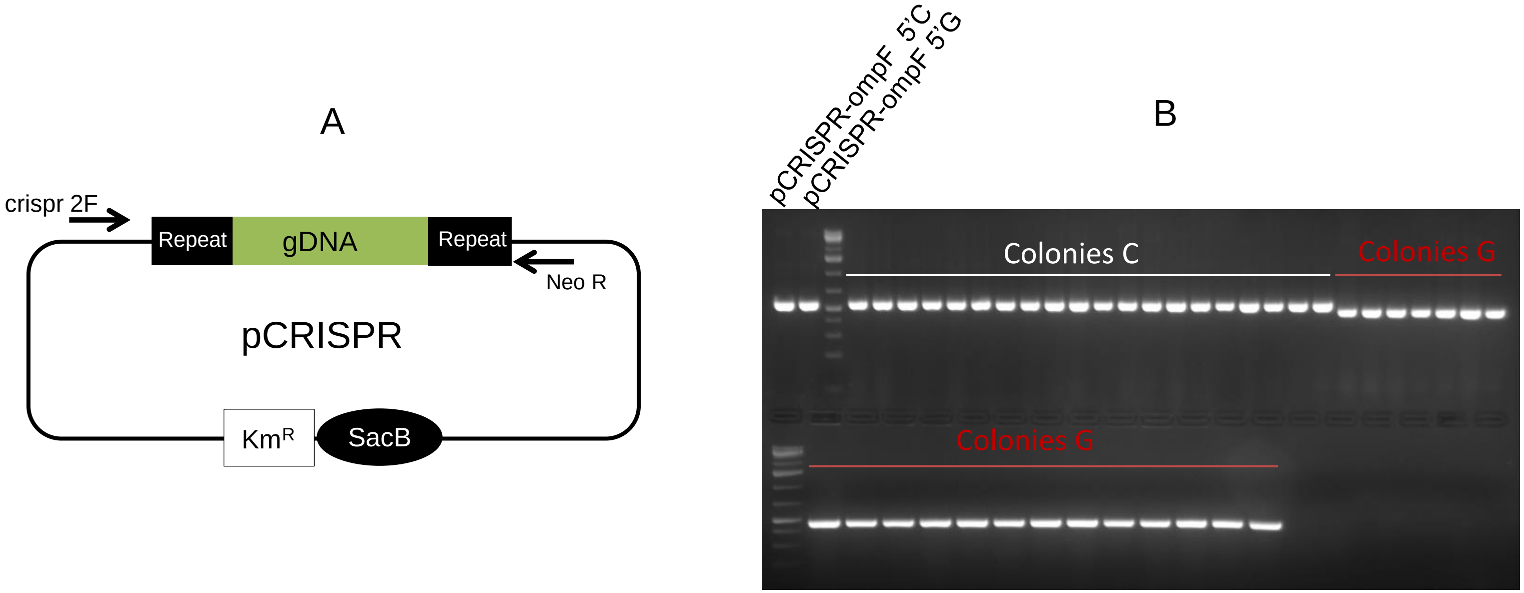

Figure S1

PCR analysis of *escapers.* (A) Schematic representation of pCRISPR-gDNA plasmid with indicated the position of the primers used to amplify the gDNA flanked by the two repeat regions. (B) Agarose gel analysis of PCR products from colonies obtained from transformation with pCRISPR-ompF 5' C (colonies C) and pCRISPR-ompF 3' G (colonies G).
